# Can the pH-dependent adsorption of phenoxyalkanoic herbicides in soils be described with a single equation?

**DOI:** 10.1007/s11356-024-35413-0

**Published:** 2024-11-08

**Authors:** Tadeusz Paszko, Claudio A. Spadotto, Miłosz Huber, Maria Jerzykiewicz, Joanna Matysiak, Alicja Skrzypek, Patrycja Boguta

**Affiliations:** 1 Department of Chemistry, University of Life Sciences, Akademicka 15, 20-950 Lublin, Poland; 2 Embrapa Digital Agriculture, Av. André Tosello, 209, Campinas, SP 13083-886 Brazil; 3 Department of Geology, Soil Science and Geoinformation, Maria Curie-Skłodowska University, Kraśnicka 2d/107, 20-718 Lublin, Poland; 4 Faculty of Chemistry, University of Wrocław, F. Joliot-Curie 4, 50-383 Wrocław, Poland; 5 Institute of Agrophysics, Polish Academy of Sciences, Doświadczalna 4, 20-290 Lublin, Poland

**Keywords:** Acidic herbicides, Adsorption, Humic substances, Soil porosity, Mineral soil profiles, Potential acidity

## Abstract

The six phenoxyalkanoic acid herbicides currently used in the European Union have similar molecular structures. Therefore, we assumed the soil components involved in the adsorption mechanisms of these herbicides to be identical. The values of the adsorption distribution coefficient* K*_*d*_, obtained via batch experiments involving typical Polish Arenosol, Luvisol, and Chernozem profiles with a native pH of 4.2–7.7, were examined using Lasso regression, as well as adsorption on isolated fractions of humic substances, Al_2_O_3_, and goethite. The neutral forms of the herbicides were adsorbed on the surface of fulvic acids available to them, covering soil mesopores with a size of > 2.5 nm. The models revealed that fulvic acids had a lognormal-like distribution in soil pores. Herbicide anions were adsorbed on the pH-dependent sites of Al oxyhydroxides and on the sites created by the Al^+3^ species adsorbed on the surface of fulvic acids (both sites were active up to pH 7.5), the sites of humic acids associated with the adsorbed Al^3+^ species, sites of Fe oxyhydroxides (active at pH < 5), and, to a limited extent, sites of humins. Two models describing the adsorption of phenoxyalkanoic acid herbicides in soils were created. A simpler model was based on humic substance fractions and the variables related to the potential acidity of soils. In the more extensive model, humic substance fractions and Al and Fe oxyhydroxide contents were used as predictors, and, where necessary, the predictors were combined with the modified Henderson–Hasselbalch formula to estimate the activity ranges of pH-dependent sorption sites. The study findings revealed that fulvic and humic acids were the main adsorbents of phenoxyalkanoic herbicides in soils, indicating that transporting of the herbicides with dissolved organic matter is an important mechanism of groundwater and surface water contamination with these chemicals.

**Supplementary Information:**

The online version contains supplementary material available at 10.1007/s11356-024-35413-0.

## Introduction

The worldwide use of phenoxyalkanoic acid herbicides (PAAHs) in agriculture adversely affects the environment. Substantial amounts of these postemergence herbicides reach the soil surface and have been frequently detected in surface water and groundwater, sometimes at concentrations that exceed permissible levels (Loos et al. 2009, 2010). Therefore, the leaching of PAAHs into groundwater should be accurately predicted in arable soil profiles. To predict pesticide leaching from the vadose zone to groundwater, several plant-, pesticide-, soil-, and climate-specific parameters, particularly adsorption parameters, should be specified in pesticide leaching programs (Jarvis 2016; Paszko and Spadotto 2022).

According to Haberhauer et al. (2000), Kah and Brown (2006), Werner et al. (2013), and Paszko et al. (2016), PAAHs that are derivatives of butyric acid (2,4-DB and MCPB) and have the highest *pK*_*a*_ (negative logarithm of the dissociation constant), log P (logarithm of the octanol/water partition coefficient), and log D (log P corrected to pH) values are more readily adsorbed into soil than acetic acid (2,4-D and MCPA) and propionic acid (DCPP-P and MCPP-P) derivatives (Table 1). Adsorption of PAAHs is negatively correlated with soil pH, and neutral and anionic forms of herbicides are adsorbed on organic matter. The contribution of soil inorganic components to the overall adsorption of PAAHs increases with soil depth. At low pH, the neutral herbicide forms can be adsorbed on quartz (Clausen et al. 2001; Paszko 2014). Anionic herbicide forms can be adsorbed on Fe and Al oxyhydroxides (Clausen and Fabricius 2001; Sannino et al. 1997), and on clay minerals such as kaolinite or calcite (Clausen et al. 2001) at higher pH. However, the interactions between soil components, including organic matter, Fe and Al oxyhydroxides, and clay minerals, considerably affect their sorption properties, which is why the overall soil adsorption usually differs substantially from combined adsorption on pure components (Celis et al. 1999; Haberhauer et al. 2000; Sannino et al. 1997).

**Table 1:**
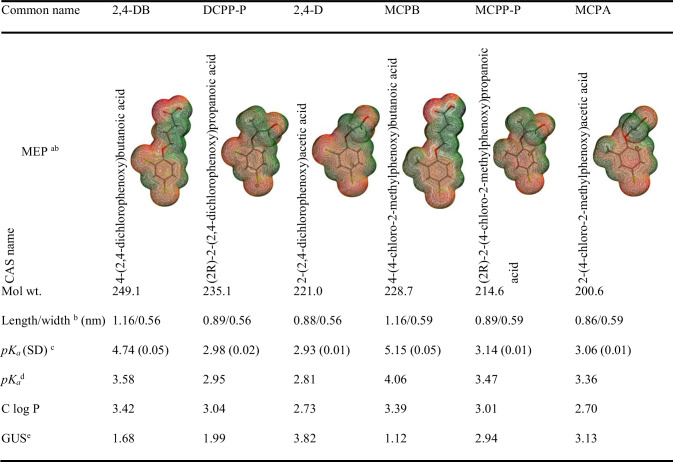
Structure and selected physicochemical properties of the examined PAAHs

^a^Molecular electrostatic potential density map (deepest green color – highest positive potential; deepest red color − highest negative potential)

^b^Calculated with SPARTAN’10 Pro (Wavefunction 2011)

^c^Determined in this study (20 °C, I = 0.01; according to Albert and Serjeant (1984))

^d^Predicted with Marvin Ver. 19.9 (ChemAxon 2024)

^e^Groundwater Ubiquity Score index (Lewis et al. 2016)

Most of the proposed models of pH-dependent adsorption of PAAHs focused on the main soil parameters (organic matter content, soil texture, Fe and Al oxyhydroxide contents) or pesticide properties such as log P and log D values, and they explained 70%–90% of adsorption variance (Franco and Trapp 2008, 2010; Kah and Brown 2007; Paszko 2014). At the beginning of the twenty-first century, considerable emphasis was placed on mitigating the environmental impact of plant protection products, which created a need for more accurate adsorption models. The pesticide leaching programs authorized by the European Union (EU) are based on the organic carbon-normalized adsorption coefficient (*K*_*OC*_) and they are not sufficiently accurate (Jarvis 2016, 2018).

Sixty of 88 synthetic herbicides that have been approved for use in the EU (as of 5 October 2024) are ionizable organic compounds, and 22 of these herbicides have Groundwater Ubiquity Score (GUS) index higher than 2.8 (Lewis et al. 2016), which indicates that they have potentially high potential for movement toward groundwater. At native soil pH, 19 of these herbicides occur mainly in anionic form, and 10 compounds are carboxylic acids. For comparison, only two of 28 non-ionic herbicides that are currently used in the EU have GUS > 2.8. These data indicate that further research should be focused on adsorption of acidic herbicides, in particular their anionic forms.

2,4-D, MCPA, and PCPP-P have GUS values > 2.8, whereas the remaining PAAHs have GUS values in the range of 1.99–1.12 (Table 1). Therefore, these herbicides were selected for adsorption studies, also because they have a similar chemical structure and occur in soil mainly in anionic form. We believe soil characteristics, including organic matter fractions, should be examined in greater detail to obtain more reliable and accurate pesticide adsorption models. To date, a limited number of studies have focused on the adsorption of PAAHs on isolated fractions of humic substances (Celis et al. 1999; Ćwieląg-Piasecka et al. 2018; Elkins et al. 2011; Haberhauer et al. 2002; Iglesias et al. 2009; Khan 1973). Research studies examining the adsorption of 2,4-D (Larrivee et al. 2003) and DCPP (Elkins et al. 2011) on fulvic acids (*FA*) after their coagulation with Al^3+^ or Pb^2+^ have shown that negatively-charged *FA* components can interact with negatively-charged herbicides through positively-charged metal ion bridges. However, the actual contribution of each humic substance fraction to the total sorption of PAAHs in soil remains unknown.

Given the structure of molecular and anionic forms of PAAHs (Table 1), the most important interactions with soil components should include specific hydrogen interactions in the carboxyl group, electrostatic interactions in a deprotonated carboxyl group, interactions with an oxygen atom in the phenoxy group, and van der Waals and hydrophobic interactions (Ćwieląg-Piasecka et al. 2018; Haberhauer et al. 2000; Prado and Airoldi 2001). The above mechanisms should occur in the six PAAHs authorized for use in the EU (EU Pesticides Database 2024). Therefore, because of the similarities in the chemical structure of the PAAHs (Table 1), we hypothesized that the same soil components should contribute to their adsorption and that these processes can be described with the same adsorption model. This study aimed to verify this hypothesis.

## Materials and methods

### Soils

Six soil profiles located in south-eastern Poland (Supplementary Fig. S1) were selected from the Database of the Polish Arable Mineral Soils (Bieganowski et al. 2013) kept by the Institute of Agrophysics of the Polish Academy of Sciences in Lublin. Two Arenosol profiles (WRB 2015), with code numbers 611 and 805 in the above database, account for 27% of Polish soils formed from sand. Luvisol profiles 590 and 824 represent 24.7% of the soils formed from loamy sand or sandy loam, Luvisol profile 564 accounts for 6.9% of the soils formed from loess and loess-like formations, whereas Chernozem profile 587 represents 1% of Polish chernozems. The Arenosol, Luvisol, and Chernozem soil groups cover 3.6%, 15%, and 2% of the land area in the European Union (Tόth et al. 2008) and 10%, 4.2%, and 1.5% of land area in the world (WRB 2015), respectively. Samples of topsoils and two subsoils were collected at a depth of 10–15 cm, 45–50 cm, and 75–80 cm, respectively. Then, the samples were air-dried, passed through a 2 mm mesh diameter sieve and homogenized.

In addition to typical soil characteristics, such as texture (*Sand*, *Silt*, *Clay* (%)), pH, and organic carbon content (*OC* (mg/g)), the following soil properties were also examined in the present study: the content of fulvic acids, humic acids, and humins (*FA*, *HA*, *HU* (mg *OC*/g)) determined with sodium pyrophosphate (Fox et al. 2017); the content of organic complexes and poorly crystallized Al and Fe oxyhydroxides (*Al(T)*, *Fe(T)* (g/kg)) determined by dissolution with the Tamm reagent (ammonium oxalate–oxalic acid) (Cave and Harmon 1997; McLaren and Crawford 1973); effective cation exchange capacity, exchangeable acidity, content of exchangeable aluminum, and hydrogen cations (*ECEC, EA, Al(EA)*, and *H(EA)* (cmol( +)/kg)) determined in barium chloride solution (ISO 11260 2018; ISO 14254 2018); potential cation exchange capacity, potential acidity, and contribution of aluminum, and hydrogen cations to potential acidity (*PCEC*, *PA*, *Al(PA)*, and *H(PA)* (cmol( +)/kg)) determined in barium chloride–triethanolamine (BaCl_2_–TEA) solution at pH 8.2 (Curtin and Rostad 1997; ISO 13536 1995); anion exchange capacity (*AEC* (cmol( −)/kg)) determined according to Wada and Okamura (1977); the specific surface area of soil (*SSA(aN2)* (m^2^/g)) determined from the Brunauer–Emmett–Teller (BET) equation based on N_2_ adsorption at 77 K; the surface area of soil pores (*SBJH(dN2)* (m^2^/g)) and the distribution of pores (with a radius of *r*_*(dN2)*_ < *1.8* to* r*_*(dN2)*_ = *30.0* nm) determined based on N_2_ desorption isotherms using the method proposed by Barrett, Joyner, and Halenda (Barrett et al. 1951).

The soil surface properties were analyzed at the Institute of Agrophysics of the Polish Academy of Sciences in Lublin. The geographic coordinates of the examined soils, their properties, and analytical methods are presented Supplementary Table S1. The remaining information can be found elsewhere (Siek and Paszko 2019; Siek et al. 2021).

The samples from the Ap horizon of profiles 587 (Chernozem, pH 7.1) and 590 (Luvisol, pH 4.5) were used to isolate *FA*, *HA* and *HU* fractions. *FA* and *HA* fractions were extracted with the use of 0.1 M Na_4_P_2_O_7_ (Audette et al. 2021; Gregor and Powell 1986; IHSS 2024), and *HU* fractions were concentrated using 2% HF (Rumpel et al. 2006). *FA* fractions were isolated with Amberlite™ XAD7HP resin from Supelco® (Gregor and Powell 1986). *HA* fractions were purified by strictly following the IHSS (2024) procedure. The applied methodology is presented in detail in Supplementary information (Section S2.1). *HU* fractions were concentrated with 2% HF to obtain the *OC* content of altered samples high enough to perform nuclear magnetic resonance (NMR) measurements and obtain spectra comparable to those of native *HU* (Rumpel et al. 2006). The freeze-dried samples were labeled as *FA*, *HA*, or *HU* with suffixes 590 Ap or 587 Ap. Some of the obtained liquid samples of *FA 587* Ap and *FA 590* Ap samples were not freeze-dried, but stored (5 °C) at their native pH ~ 2.8 and later used for adsorption experiments.

The content (mg/g) of *OC*, nitrogen, sulfur and hydrogen in the *FA*, *HA*, and *HU* fractions (Supplementary Table S2) was determined using a Vario El cube CHNS elemental analyzer (Elementar, Langenselbold, Germany). The ^13^C NMR spectra of the isolated humic substances were obtained by CP/MAS 13C-NMR spectroscopy. The analysis was performed in a Bruker AVANCE III NMR device (Brucker Co., Billerica, MA, USA) in the Faculty of Chemistry of the University of Wrocław. Details are presented in Supplementary information (Section S2.2).

The surface of sand grains was examined under a Hitachi SU6600 scanning electron microscope with an EDS attachment (Thermo Fisher Scientific Inc., Waltham, MA, USA). The surface of *HU* was examined under a Quanta 3D FEG scanning electron microscope (Thermo Fisher Scientific Inc.) with an EDX Octane Elect Plus attachment (AMETEK Instruments India Pvt Ltd., Bangalore, India). Details are presented in Supplementary information (Section S2.3). The analyses were carried out in the Department of Geology, Soil Science, and Geoinformation and the Analytical Laboratory at the Chemical Science Institute of the Maria Curie–Skłodowska University in Lublin.

### Materials and chemicals

Methanol stock solutions (250 mg/L) of the analyzed herbicides were prepared using the certified analytical standards (99.9% ± 0.1%, Sigma-Aldrich, Poznań, Poland; or 99.7%–99.8% ± 0.1%, Institute of Organic Industrial Chemistry, Warsaw, Poland) and high-performance liquid chromatography (HPLC)-grade methanol. The remaining chemicals and solvents used in this study were of analytical or HPLC grade. Aqueous solutions of PAAHs and other aqueous solutions were prepared from the stock solutions using ultrapure water with a conductivity of 0.05 µS/cm. Commercial goethite (α-FeOOH) was purchased from Sigma-Aldrich (Poznań, Poland). Al_2_O_3_ (γ-aluminum; 20 nm, > 99% purity) was purchased from IoLiTec Ionic Liquids Technologies (Heilbronn, Germany). The Spectra/Por® 6 Dialysis Tubing Membrane (diameter: 11.5 mm, MWCO: 1 kD) was purchased from Carl Roth GmbH + Co. (Karlsruhe, Germany).

### Adsorption on soils, goethite, Al_2_O_3_, and HU fractions

The batch adsorption experiments were conducted according to the OECD Guideline 106 (OECD 2000). To obtain an equilibrium concentration of PAAHs in the aqueous phase at least of  > 10% of their initial concentration, and preferably > 50% (OECD 2000), the applied soil:solution ratio was 1:5 for 2,4-DB and MCPB and 1:1.5 for 2,4-D, MCPA, DCPP-P, and MCPP-P. The initial concentration of each PAAH in 0.01 M CaCl_2_ and 5·10^−5^ M HgCl_2_ (biocide; the concentration used did not affect the adsorption of PAAHs; see Table S3) was 3.0 mg/L. The triplicate samples (and duplicate control and blank samples) were agitated for 24 h on a rotator (20 rpm; 20 °C ± 0.5 °C). At the end of agitation, the pH of soil suspensions was measured at 20 °C ± 0.5 °C with a glass electrode, the tubes were centrifuged (20 min, 3300 g, 20 °C ± 1 °C), and the liquid phase was sampled for HPLC analysis. Adsorption was calculated from the difference between the initial concentration and the concentration after 24 h (for more details, see Supplementary information Subsection S2.4).

The methods for analyzing the adsorption of PAAHs on goethite, Al_*2*_O_3_, *HU* fraction, and *HU* fraction altered by the adsorption of Al^3+^ were based on the OECD Guideline 106 (OECD 2000). However, different adsorbent:solution ratios and different initial concentrations of PAAHs were used in the experiments. The applied modifications are presented in detail in Supplementary information (Sections S2.4. and S2.5). The difference was also that the pH of the suspensions of the above adsorbents was adjusted to five pH levels (from ~ 2.9 to ~ 5.5 for goethite; and from ~ 3 to ~ 7 for Al_2_O_3_ and the *HU* samples) with NaOH. The adsorption of Al^3+^ on *HU* fraction was performed by pipetting AlCl_3_ solutions into the *HU* samples at concentrations that would lead to the adsorption of 0.09, 0.35, 0.87, or 1.74 mmol( +) Al^3+^/g *OC* (on the assumption that Al^3+^ would be adsorbed in 100%). The amount of 0.09 mmol( +) Al^3+^/g *OC* is the average amount obtained from Eq. (8) for 18 soils, and subsequent values denote fourfold, tenfold and 20-fold multiples of that amount.

### Adsorption on FA and HA fractions

The experiments analyzing the adsorption of PAAHs on *FA* were performed using the equilibrium dialysis method (De Paolis and Kukkonen 1997). The Spectra/Por® 6 Dialysis Tubing Membrane was cut into 7-cm pieces, soaked for 30 min in ultrapure water, thoroughly rinsed to remove preservatives, and one side of each membrane was closed with a clip. The *FA* solution was pipetted into volumetric flasks, which were placed on a magnetic stirrer, and the pH of the solutions was gradually adjusted to five values (from ~ 3 to ~ 7.5) with using 0.3 M NaOH. The solution in each flask was diluted to the same volume using ultrapure water. The content of *FA* in the *FA* 587 Ap and *FA* 590 Ap solutions with adjusted pH was determined at 0.654 and 0.468 mg/mL, respectively. The *FA* solutions were dosed (2 mL) into membranes with a known moisture content (calculated from the weight difference). The membranes were closed with a second clip and placed inside polypropylene test tubes. Next, 0.025 M CaCl_2_ solution with 0.0125% NaN_3_ (biocide) was dosed (2 mL) into test tubes, and 25 mg/L of the PAAH solution was added (1 mL). The apparent initial concentration of herbicides in the duplicate test tubes was 5 mg/mL. Finally, the test tubes were placed in the rotator and slowly agitated (2 rpm, 120 h, 20 °C ± 0.5 °C). Previous experiments conducted by De Paolis and Kukkonen (1997) revealed that 72 h was sufficient for xenobiotics to pass the Spectra/Por® 6 Dialysis Tubing Membrane with MWCO of 1 kD and achieve adsorption equilibrium. After agitation, membranes were removed from test tubes, the pH of the solutions in the test tubes was measured, the tubes were centrifuged (20 min, 3300 g, 20 °C ± 1 °C), and the liquid phase was sampled for HPLC analysis. Two series of adsorption experiments were conducted. All PAAHs and two *FA* were tested in the first series at two pH values (~ 2.9 (native) and ~ 5.1). In the second series, the selected PAAHs and one *FA* were tested at five pH values (~ 3 to ~ 7).

The adsorption experiments were also performed using *FA* suspensions with adsorbed Al^3+^ species. Initially, the selected *FA* solution was pipetted into the volumetric flasks, and specific volumes of the AlCl_3_ solution were added to induce the adsorption of up to 5.12, 20.47, 51.18, or 102.37 mmol( +) Al^3+^/g *OC* (the arithmetic mean for 18 soils from Eq. (8) for the *FA*_>*2.5L*_ variable and its fourfold, tenfold and 20-fold multiples, respectively). Next, the pH of the suspensions was slowly adjusted to five values (from ~ 4 to ~ 7.5) using NaOH. The next steps of these experiments were similar to those described in the *FA* analysis, and details are presented in Supplementary information (Section S.2.6). At each of the five pH values, the duplicate samples of the three selected PAAHs and one of the tested *FA* (with the lowest content of Al^3+^, which significantly increased herbicide adsorption) were used in this series of adsorption experiments. The adsorption of the duplicate samples of all PAAHs in the *FA* 587 Ap and *FA* 590 Ap suspensions (with the mentioned content of Al^3+^) was analyzed at pH ~ 5.1.

The experiments analyzing the adsorption of PAAHs on *HA* followed a similar protocol to that described in the *FA* analysis. The applied methodology is presented in detail in Supplementary information (Section S2.6). In the following series of adsorption experiments, AlCl_3_ was added to *HA* suspensions, and next the adsorption of PAAHs was carried out. AlCl_3_ solutions were used to induce the adsorption of up to 0.16, 0.64, 1.61, or 3.21 mmol( +) Al^3+^/g *OC*. The lowest value was obtained for the *HA* variable from Eq. (29) or (30), and subsequent values were the fourfold, tenfold, and 20-fold multiples of that value. Details are presented in Supplementary information (Section S2.6).

### Determination of pK_a_ and HPLC measurements

The *pK*_*a*_ values of PAAHs (Table 1) were determined experimentally at a temperature of 20 ± 0.5 °C and the ionic strength of 0.01 M using the spectrophotometric method described by Albert and Serjeant (1984). The procedure was described in detail by Paszko et al. (2020). *pK*_*a*_ was measured because the *pK*_*a*_ values of the PAAHs differ considerably in online databases and the literature sources (refer to Table 1 by Paszko et al. (2016)). The exemplary calculated values of *pK*_*a*_ (Marvin Ver. 19.9 (ChemAxon 2024)) are presented in Table 1.

HPLC measurements were performed in a Waters HPLC device (Waters Corporation, Milford, MA, USA) using a Thermo Scientific BDS Hypersil C18 column (Thermo Fisher Scientific Inc.). Details are presented in Supplementary information (Section S.2.7).

### Modeling of pH-dependent adsorption

Assuming that both the neutral and anionic forms of the PAAHs are adsorbed on *FA*, *HA* and *HU* fractions of humic substances, the pH-dependent adsorption model can be described as follows (Jafvert 1990; Kah and Brown 2006; Paszko 2014; Spadotto and Hornsby 2003):

1 $${K}_{d}={\Phi }_{n}(FA {\kappa }_{FA.n}+HA {\kappa }_{HA.n}+HU {\kappa }_{HU.n})+{\Phi }_{an}(FA {\kappa }_{FA.an}+HA {\kappa }_{HA.an}+HU {\kappa }_{HU.an}),$$

where *κ*_*FA.n*_, *κ*_*HA.n*_,* κ*_*HU.n*_,* κ*_*FA.an*_,* κ*_*HA.an*_, and* κ*_*HU.an*_ are the regression coefficients that describe the maximum adsorption of neutral form the PAAHs (*Φ*_*n*_ = 1) and the maximum adsorption of anionic form PAAHs (*Φ*_*an*_ = 1) by the respective fractions. The *Φ*_*n*_ and *Φ*_*an*_ terms are the fractions of neutral and anionic forms of a monovalent acidic herbicide, that have been calculated as a function of *pK*_*a*_ and the actual pH in the soil suspension with the use of the modified Henderson–Hasselbalch equation:

2 $${\Phi }_{n}={~}^{1}\!\left/ \!{~}_{(1+{10}^{pH-{pK}_{a}})}\right.$$

3 $${\Phi }_{an}=1-{\Phi }_{n}$$

*Φ*_*n*_ and *Φ*_*an*_ express the nonlinear relationship between *K*_*d*_ and soil components that contribute to adsorption, which is significant when the pH of the examined soil is within the range of the herbicide *pK*_*a*_ ± 2. Equation (1) assumes the existence of a linear relationship between the amount of adsorbed PAAH and its equilibrium concentration in an aqueous solution. The reason is that the concentration-dependent changes in the adsorption of ionizable organic compounds in soil are usually negligible compared to the effect of pH.

In addition to pH-dependent changes in neutral and ionized forms of the herbicides, the number of soil sorption sites is determined by pH. Organic matter contains a large number of carboxyl and phenolic groups. Considering the acidic sorption sites of organic matter, the pH-dependent changes of their neutral ${\mathrm{f}}_{\mathrm{n}}$ and anionic forms ${\mathrm{f}}_{\mathrm{an}}$ can be described as

4 $${\mathrm{f}}_{n}={~}^{1}\!\left/ \!{~}_{(1+{10}^{(pH-{pK}_{a}^{OM})\!\left/{\eta }\right.})}\right.$$

5 $${\mathrm{f}}_{an}=1-{\mathrm{f}}_{n}$$

It is generally assumed that the negative logarithm of the mean dissociation constant of organic matter ${pK}_{a}^{OM}$ equals 5, and the empirical value *η* equals 2 (McBride 1995; Spadotto and Hornsby 2003). Equations (4) and (5) can also be used to model the number of neutral and positive sorption sites of organic matter induced by its amine groups, as well as the number of neutral or positive sorption sites of quartz and silica, or Fe and Al oxyhydroxides. In the above case, ${\mathrm{f}}_{n}$ in Eq. (4) is denoted by ${\mathrm{f}}_{p}$, and ${\mathrm{f}}_{an}$ in Eq. (5) is denoted by ${\mathrm{f}}_{n}$.

When three or more soil components adsorb the neutral and anionic forms of the herbicide, the collinearity of independent variables increases considerably, and correct values of regression coefficients and their significance levels are difficult to estimate using simple linear or nonlinear regression. For example, the terms in Eq. (1) are strongly correlated because each fraction of humic substances is multiplied by *Φ*_*n*_ or by *Φ*_*an*_, and because *FA* + *HA* + *HU* = *OC*. Therefore, adsorption was modeled using Lasso regression, which copes with collinearity by combining the least squares method with an effective penalty formula based on the parameter λ which is optimized during cross-validation (Zumel and Mount 2019). The analyses involved matrices of independent variables *X*_*i*_* Φ*_*n*_ and *X*_*i*_* Φ*_*an*_, which were created for soil *i* by multiplying soil component/property *X* by *Φ*_*n*_ or *Φ*_*an*_. During the Lasso regression procedure, λ_optimal_ values were estimated using, as a rule, tenfold cross-validation (Zumel and Mount 2019). One of the advantages of Lasso regression is that the regression coefficients for the significant independent variables are optimized during cross-validation, whereas nonsignificant variables are automatically eliminated from the model. For some calculations, Ridge regression was used because, unlike Lasso regression, it generally assumes that all independent variables play an important role in explaining the dependent variable, and the main task of the penalty function is to optimize the regression coefficients (Zumel and Mount 2019).

Lasso and Ridge regressions were carried out using the R Glmnet package (Zumel and Mount 2019) combined with XLSTAT 2023.1.2 software (Lumivero, Denver, CO, USA) (Lumivero 2023). The remaining statistical analyses were performed in Statistica 14.0.0.15 (TIBCO Software Inc., Palo Alto, CA, USA).

## Results and discussion

### Adsorption of PAAHs in soils

#### Preliminary analyses

The determined values of *K*_*d*_ and the corresponding pH values in the soil suspensions are presented in Supplementary Table S4. The mean *K*_*d*_ values for 18 topsoils and subsoils, arranged in descending order, were as follows: 3.06 mL/g (2,4-DB), 2.43 mL/g (MCPB), 0.33 mL/g (2,4-D), 0.27 mL/g (MCPA), 0.24 mL/g (DCPP-P), and 0.17 mL/g (MCPP-P). Adsorption was highest for butyric acid derivatives, lower for acetic acid derivatives, and lowest for propionic acid derivatives. Moreover, adsorption was somewhat higher for derivatives with the 2,4-dichlorophenoxy group and lower for derivatives with the 4-chloro-2-methylphenoxy group, which is characteristic of PAAHs (Paszko et al. 2016). The obtained *K*_*d*_ values were below or within the lower range of values from the EU dossiers presented in the Pesticide Properties Database (Lewis et al. 2016)), which can be attributed to the fact that Polish soils are generally low in *OC*. The median *OC* values for examined topsoils and subsoils were 1.10% and 0.13%, respectively.

**Fig. 1:**
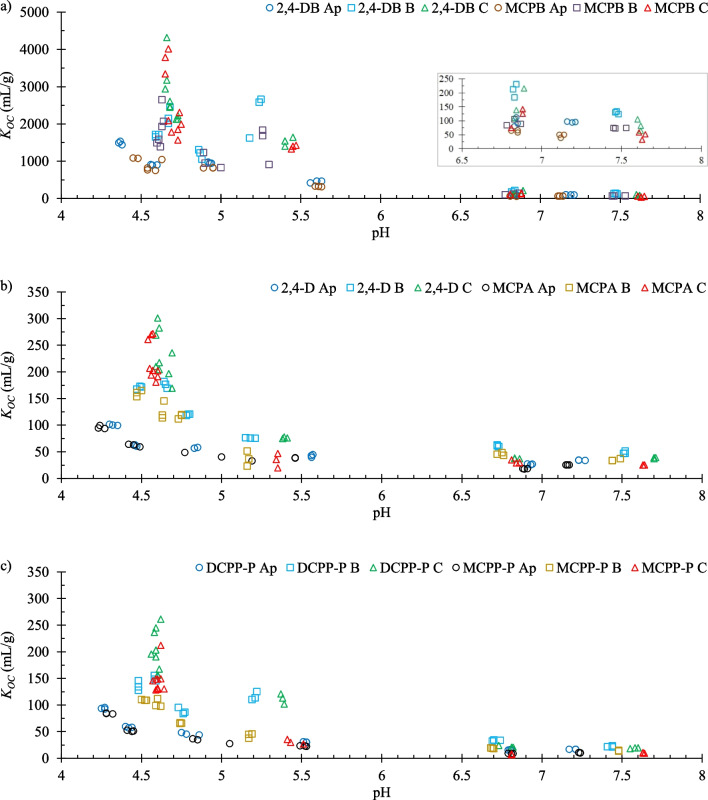
Relationship between *K*_*OC*_ and pH for (**a**) 2,4-DB and MCPB, (**b**) 2,4-D and MCPA, (**c**) DCPP-P and MCPP-P for topsoil (Ap) and two subsoil levels (B and C)

PAAHs are adsorbed mainly by organic matter, which is why *K*_*OC*_ values were calculated for the preliminary analysis. Much greater variance in the *K*_*OC*_ values for each herbicide was noted at pH of < 5.5 than at pH of > 5.5 (Fig. 1). At pH of < 5, *K*_*OC*_ values were lowest for topsoils (Ap), somewhat higher for upper subsoils (B), and much higher for lower subsoils (C), which suggests that inorganic soil components made a certain contribution to the adsorption. Moreover, *K*_*OC*_ values were somewhat higher at a pH of 5.2–5.6 than 6.7–7.8 (Fig. 1bc). The above suggests that some 2,4-D, MCPA, DCPP-P, and MCPP-P anions, and probably also 2,4-DB and MCPB anions, were adsorbed on soil components whose sorption sites became inactive at higher pH. The data presented in Fig. 1 suggested that inorganic soil components contributed to adsorption, which is why *K*_*d*_ values were used in further analyses.

**Fig. 2:**
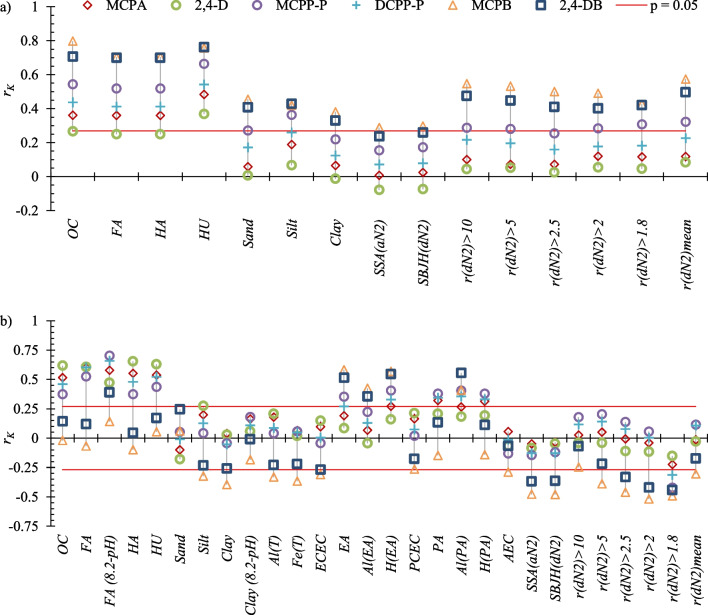
Values of the Kendall’s correlation coefficients *r*_*K*_ showing the relationship between *K*_*d*_ and transformed *X*_*i*_ variables (**a**) *X*_*i*_* Φ*_*n*_ or (**b**) *X*_*i*_* Φ*_*an*_ presented on the X-axis

The preliminary correlation analysis was based on Kendall’s rank correlations (most variables were not normally distributed). The correlations between *K*_*d*_ vs. *X Φ*_*n*_ and *X Φ*_*an*_ were analyzed to distinguish between the adsorption of neutral and anionic forms of the herbicide on the *X* soil component. The presence of significant positive correlations suggested that neutral forms of PAAHs were adsorbed predominantly on *FA*, *HA*, and *HU* fractions (Fig. 2a**)**. Moreover, the *K*_*d*_ values for some herbicides were significantly positively correlated with *Sand Φ*_*n*_ and *Silt Φ*_*n*_. The values of *K*_*d*_ were also positively correlated with soil pore size distribution.

The analysis concerning the adsorption of anionic forms of herbicides (Fig. 2b) revealed the strongest correlations for *OC* and humic substance fractions. Moreover, strong positive correlations were observed with *EA Φ*_*an*_, *H(EA) Φ*_*an*_, and *Al(PA) Φ*_*an*_, which suggests that exchangeable or extractable H^+^ and Al^3+^ cations contributed to the adsorption of PAAH anions. In the analysis of *FA* and *Clay* variables, *X*_*i*_* (8.2-pH) Φ*_*an*_ expressions were tested in addition to *X*_*i*_* Φ*_*an*_. The term *(8.2-pH)* describes the pH-dependent changes in the amount of sorption sites responsible for the soil potential acidity. This term was taken from the regression model (*PA* = b + a_1_ (8.2-pH) *FA* + a_2_ (8.2-pH) *Clay*) developed by Curtin and Rostad (1997). *K*_*d*_ was more strongly correlated with *FA (8.2-pH) Φ*_*an*_ than with *FA Φ*_*an*_, which suggested that PAAH anions were adsorbed on pH-dependent sorption sites of the *FA* fraction associated with *PA* (i.e., the sites associated with extractable H^+^ and Al^3+^). No significant correlations were noted for *Clay Φ*_*an*_ or *Clay (8.2-pH) Φ*_*an*_.

Because the strongest correlations were noted for *OC* and the *FA*, *HA*, and *HU* fractions (Fig. 2), these variables were examined in the first two steps of the Lasso regression. The values of the adjusted coefficient of multiple determination (R_a_^2^) were not very high (0.737–0.912) in the models with *OC* (Supplementary Eqs. (S1)–(S3) and (S19)–(S21) in Supplementary Table S7), and in the models with *FA*, *HA*, and *HU* fractions (Supplementary Eqs. (S4)–(S6) and (S22)–(S24)) were only somewhat higher (range 0.867–0.928). In the following two steps, inorganic soil components were analyzed with *OC* or *FA*, *HA*, and *HU*. The values of R_a_^2^ in models with *OC* (Supplementary Eqs. (S7)–(S9) and (S25)–(S27)) ranged from 0.896 to 0.961, but were noticeably higher (0.958–0.977) in models with humic substances fractions (Supplementary Eqs. (S10)–(S12) and (S28)–(S30)). Supplementary Eqs. (S10)–(S12) and (S28)–(S30) suggested that *HU*, *Sand*, and *FA* were responsible for the adsorption of neutral forms of PAAHs, whereas the adsorption of their anionic forms was associated with the *HA*, *HU*, *Al(PA)*, and *H(PA)* variables. However, the models were inconsistent, and the large number of predictors was questionable. In addition, the data presented in Fig. 2a suggested that soil porosity could also affect adsorption, and this possibility has not been explored to date.

#### Impact of FA distribution in soil pores on adsorption

Two types of humic substances have been distinguished in conceptual models describing the interactions between hydrophobic organic contaminants and soil particles (Bogan et al. 2003; Xing and Pignatello 1997). The first type, comprising *FA* and *HA*, has a “soft”, gel-like colloidal nature. The second type is comprised rigid, inflexible, “glassy” or “hard” *HU*, which constitute the predominant type of humic substance that, alongside clay minerals, forms the core of soil particles. According to Bogan et al. (2003) and Ravikovitch et al. (2005), “soft” *FA* and *HA* are bound or complexed on the external surface of clay minerals and “glassy” *HU*. The content of these fractions on the surface and the mean thickness of the “soft” layer can be estimated by dividing the content of *FA* and *HA* (mg/g) by the surface area (m^2^/g) obtained from the BET N_2_ isotherm at 77 K.

Soil pores with small radii were widely distributed in the examined soils (e.g., 4.4%–13.3% for 1.5–1.8 nm micropores, 18.1%–50.6% for 1.8–2.0 nm micropores, or 13.8%–22.2% for 2.0–2.5 nm mesopores; Supplementary Table S1), and the length and width of the examined PAAHs were determined at 0.86–1.16 nm and 0.56–0.59 nm, respectively (Table 1; SPARTAN’10 Pro (Wavefunction 2011)). This suggested that part of the “soft” layer bound to the surface of the smallest pores was inaccessible for PAAHs. Therefore, the *FA* content (mg/g) was calculated based on the known *SBJH* values for pores with *r*_*(dN2)*_ of > 1.8, > 2.0, > 2.5, > 3.5, > 5.0, and > 10.0 nm (denoted as *FA*_>*1.8C*_, *FA*_>*2.0C*_, *FA*_>*2.5C*_, *FA*_>*3.5C*_, *FA*_>*5.0C*_, and *FA*_>*10.0C*_; the letter “*C*” in the subscript indicates that the layer on the surface of soil pores exhibited a fairly constant thickness). Similar calculations were performed for *HA*, and the obtained variables were used in modeling. The calculations are presented in detail in Supplementary Table S8.

New *FA*_>*1.8C*_ − *FA*_>*10.0C*_ variables were applied in the modeling process, which increased the prediction of *K*_*d*_ and reduced the number of independent variables. The values of R_a_^2^ increased when these variables were attributed to the adsorption of the neutral form of PAAHs. In the model where *FA* or one of the above variables were responsible for adsorbing the neutral form of PAAHs and where the *HA*, *HU*, *Al(PA)*, and *H(PA)* variables were responsible for adsorbing the respective anions, the mean R_a_^2^ values for the six analyzed PAAHs were as follows: 0.9379 for FA, 0.9641 for *FA*_>*2.0C*_, 0.9717 for *FA*_>*2.5C*_, 0.9723 for *FA*_>*3.5C*_, 0.9621 for *FA*_>*5.0C*_, and 0.9379 for *FA*_>*10.0C*_. The value of R_a_^2^ was the highest for *FA*_>*3.5C*_; therefore, this variable was selected for further modeling (Supplementary Table S7). In turn, the replacement of *HA* with one of the new variables (*HA*_>*1.8C*_ − *HA*_>*10.0C*_) in Supplementary Eqs. (S13)–(S15) and (S31)–(S33) did not improve the prediction.

The observation that *FA* adsorbed in pores with *r*_*(dN2)*_ of < 3.5 nm were unavailable for PAAHs, whereas the entire *HA* were available, is not fully consistent with the conceptual model proposed by Bogan et al. (2003). A probable explanation was that *FA* are predominant in micropores and mesopores with the smallest radii, whereas *HA* are predominant in the larger mesopores. The molecular weight is in the range of 1000–5000 for *FA*, 10 000–100 000 for *HA*, and > 100 000 for *HU* (McBride 1995; Stevenson 1994); therefore, *FA* colloids easily diffuse into the smallest pores. Humic substances with the lowest molecular weight can be adsorbed even in the interlamellar spaces of expandable layer silicates, including montmorillonite or vermiculite (Kodama and Schnitzer 1971; Stevenson 1994).

**Fig. 3:**
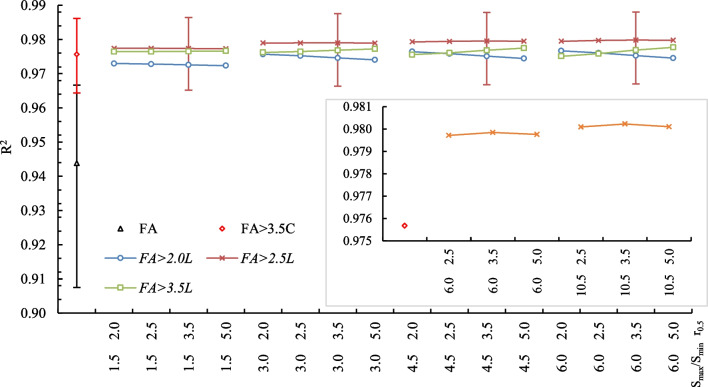
Mean R^2^ values for six PAAHs for the Lasso regression model ${K}_{d}={\Phi }_{n}FAx {\kappa }_{FAx.n}+{\Phi }_{an}(HA {\kappa }_{HA.an}+HU {\kappa }_{HU.an}+Al(PA) {\kappa }_{Al\left(PA\right).an}+H(PA) {\kappa }_{H(PA).an})$. The *FAx* variables were calculated with the use of Eq. (6) for different S_max_/S_min_ ratios and r_0.5_ values; the results for the *FA* and *FA*_>*3.5C*_ variables are presented for comparative purposes. Selected error bars – minimum and maximum

The modified logistic function was used to approximate the distribution of *FA* in soil pores:

6 $${S}_{{r}_{i}}={S}_{min}+\frac{{S}_{max}}{{2}^{{\left(\frac{{r}_{i}}{{r}_{0.5}}\right)}^{2}}}$$

where *S*_*min*_ and *S*_*max*_ (mg/m^2^) are the minimum and maximum contents of *FA* in pores, ${S}_{{r}_{i}}$ (mg/m^2^) is the content of *FA* in pores with radius *r*_*i*_, and *r*_*0.5*_ is the radius of pores with *FA* content of (*S*_*max*_ − *S*_*min*_)/2. This simple function, where each parameter has a clear meaning, produces sigmoid-shaped lines with lognormal and exponential distributions. For each soil, the ${S}_{{r}_{i}}$ values were fitted for pores with* r*_*(dN2)*_ of 1.8, 2.0, 2.5, 3.5, 5.0, and 30.0 nm by minimizing the term ${(FA-{\sum }_{i=1}^{n}{S}_{{r}_{i}})}^{2}$. The *S*_*max*_* /S*_*min*_ ratios of 1.5, 3.0, 4.5, 6, 7.5, 9.0, and 10.5 and *r*_*0.5*_ values of 2.0, 2.5, 3.5, and 5.0 nm were used. The values of *FA*_>*2.0L*_, *FA*_>*2.5L*_*,* and *FA*_>*3.5L*_ (where *L* denotes lognormal like distribution) were calculated in the next step, and the obtained variables were applied in Lasso modeling. The highest mean values of R^2^ (coefficient of multiple determination) were obtained for *FA*_>*2.5L*_ (Fig. 3). This implies that PAAHs diffusion and adsorption in pores with *r*_*dN2*_ of < 2.5 nm are very difficult. The mean R^2^ values in the models with *FA*_>*2.5L*_ and different *S*_*max*_* /S*_*min*_ ratios were the highest at *r*_*0.5*_ of 3.5 nm, i.e., were 0.9790 at a ratio of 3.0, 0.9796 at a ratio of 4.5, 0.9798 at a ratio of 6.0, and 0.9802 at a ratio of 10.5. Thus, *r*_*0.5*_ = of 3.5 nm and *S*_*max*_* /S*_*min*_ ratio ~ 3 to 6 appear to be the optimal parameters for Eq. (6).

The mean differences in the explained variance of *K*_*d*_ between the models with *FA* and *FA*_>*3.5C*_ (3.2%) and the models with *FA* and *FA*_>*2.5L*_ (3.6%; *r*_*0.5*_ = 3.5 nm and *S*_*max*_*/S*_*min*_ = 6) were not very high (Fig. 3), but an increase in prediction was observed for all PAAHs. Most importantly, these variables supported the development of a consistent model with the same independent variables for all PAAHs. Therefore, *r*_*0.5*_ = 3.5 nm and* S*_*max*_*/S*_*min*_ = 6 were used in further analyses for *FA*_>*2.5L*_ as a variant approximating maximum nonlinear of *FA* distribution in soil pores. The model with *FA*_>*3.5C*_ was simultaneously tested as a variant with uniform *FA* distribution. In this stage of research, the following model was obtained for the six tested PAAHs:

7 $${K}_{d}={\Phi }_{n}{FA}_{X} {\kappa }_{{FA}_{X}.n}+{\Phi }_{an}\left(HA {\kappa }_{HA.an}+HU {\kappa }_{HU.an}+Al\left(PA\right){\kappa }_{Al\left(PA\right).an}+H\left(PA\right){\kappa }_{H\left(PA\right).an}\right),$$

where either *FA*_>*3.5C*_ or *FA*_>*2.5L*_ can be used as *FA*_*X*_.

#### Adsorption of PAAH anions associated with potential soil acidity

The adsorption associated with *Al(PA)* and *H(PA)* explained a significant part of *K*_*d*_ variance for PAAHs in Eq. (7) (1.7% − 21.9%, mean value 14.0% for Eq. (7) with *FA*_>*3.5C*_ or *FA*_>*2.5L*_). Therefore, attempts were made to identify soil components associated with these sorption sites. The values of correlation coefficients were higher when the soil components potentially associated with *Al(PA)* and *H(PA)* were multiplied by the term *(8.2-pH)*. These results could be attributed to the research methodology (Curtin and Rostad 1997), where *Al(PA)* and *H(PA)* were extracted from soil with the BaCl_2_–TEA solution with pH 8.2. The values of Pearson’s correlation coefficient *(r*; *p*-values are not shown because most variables did not have normal distribution) suggested that variables (8.2-pH) *FA*_>*2.5L*_ (*r* = 0.885) or (8.2-pH) *FA*_>*3.5C*_ (*r* = 0.884), (8.2-pH) *Sand* (*r* = 0.752), and (8.2-pH) *HU* (*r* = 0.618) were potentially associated with *Al(PA)*. In the best Lasso models,

8 $$Al\left(PA\right)=\left(8.2-pH\right) \left(0.1954 {FA}_{>2.5L}^{\left(0.54\right)}+0.0008 {Sand}^{\left(0.48\right)}+0.0033 {HU}^{\left(0.13\right)}\right)$$

9 $$Al\left(PA\right)=\left(8.2-pH\right) \left(0.1266 {FA}_{>3.5C}^{\left(0.51\right)}+0.0008 {Sand}^{\left(0.50\right)}+0.0039 {HU}^{\left(0.16\right)}\right)$$

R_a_^2^ was determined at 0.930 and 0.926, and λ_optimal_ was determined at 0.0031 and 0.0029, respectively. In Eqs. (8) and (9), the superscript values in brackets are the standardized regression coefficients that approximate the contribution of individual soil components to *Al(PA)*.

Al^+3^ species are effectively adsorbed by *FA* because this fraction of humic substances contains the largest number of carboxyl groups. The concentration of carboxyl groups was lowest in *HU* (Supplementary Table S14), but in the examined soils *HU* content was 16 times higher on average than the content of *FA*_>*3.5C*_ and 20 times higher than the content of *FA*_>*2.5L*_. The high standardized regression coefficients for the (8.2-pH) *Sand* variable in Eqs. (8) and (9) suggested that in acidic soils, especially soils with high sand content and very low silt and clay content, sand can be the predominant inorganic adsorbent of Al^3+^ species. The surface of quartz sand grains, examined under a scanning electron microscope with an EDS attachment (Supplementary Table S15; see also Supplementary Figs. S3 and S4), was covered by illite, Al (gibbsite) and Fe (goethite) oxyhydroxides, and fine plaques of muscovite and biotite. Most importantly, the analysis of the chemical composition of sand surface revealed high Al concentration of 4.4 ± 2.5% (standard deviation).

*H(PA)* was strongly positively correlated with (8.2-pH) *Silt* (*r* = 0.629), (8.2-pH) *Clay* (*r* = 0.759), (8.2-pH) *Silt* + *Clay* (*r* = 0.808), (8.2-pH) *Al(T)* (*r* = 0.939), and (8.2-pH) *Fe(T)* (*r* = 0.875). In the group of humic substance fractions, the strongest correlations were found between *H(PA)* and (8.2-pH) *FA*_>*2.5L*_ (*r* = 0.352) or (8.2-pH) *FA*_>*3.5C*_ (*r* = 0.324). The values of R_a_^2^ were identical (0.980) in both Lasso regression models (λ_optimal_ was 0.074 and 0.024, respectively):

10 $$H\left(PA\right)=\left(8.2-pH\right) \left(0.016 {\left(Silt+Clay\right)}^{\left(0.33\right)}+1.481 {Al\left(T\right)}^{\left(0.45\right)}+0.050 {Fe\left(T\right)}^{\left(0.12\right)}+0.856 {FA}_{>2.5L}^{\left(0.19\right)}\right)$$

11 $$H\left(PA\right)=(8.2-pH)\left(0.017 {\left(Silt+Clay\right)}^{\left(0.34\right)}+1.379 {Al\left(T\right)}^{\left(0.42\right)}+0.064 {Fe\left(T\right)}^{\left(0.16\right)}+0.643 {FA}_{>3.5C}^{\left(0.21\right)}\right)$$

Eqs. (10) and (11) are fully consistent with the results obtained by other authors, which indicate that extractable acidity is directly associated with hydrogen cations that are adsorbed on clay and silt fractions and humic substances (in particular on the *FA* fraction) (Curtin et al. 1987; Curtin and Rostad 1997), and, indirectly, with Al^3+^ and (to a limited extent) Fe^3+^ species which are hydrolyzed after desorption with BaCl_2_–TEA solution, which increased the concentration of H^+^ in BaCl_2_–TEA (Bai et al. 2018; Curtin et al. 1987; McBride 1995).

**Fig. 4:**
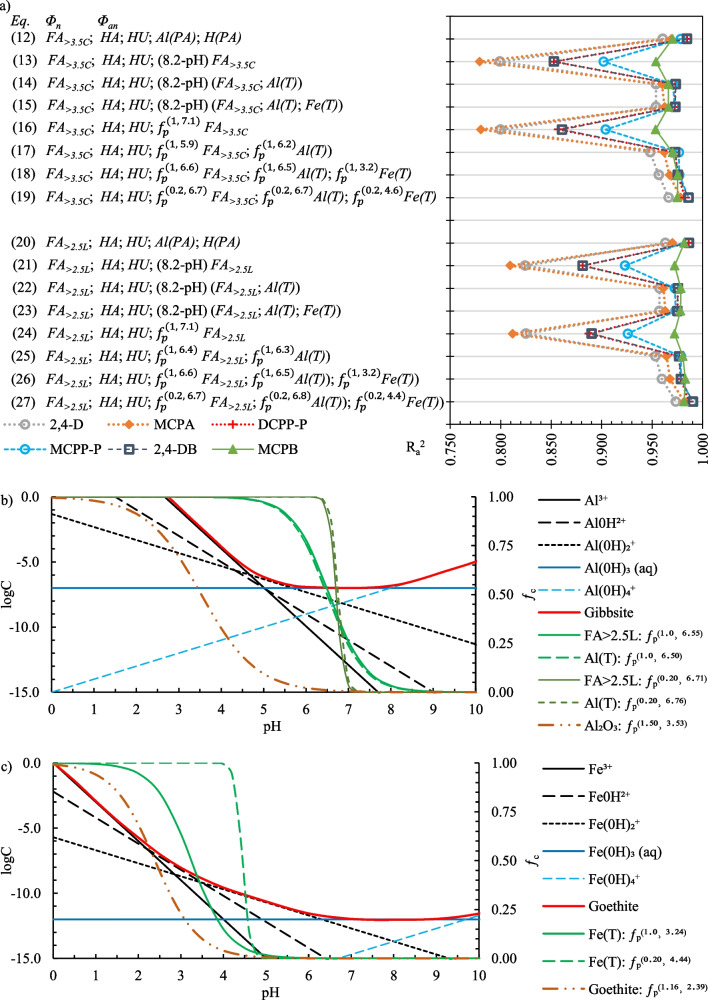
R_a_^2^ values for the Lasso regression models on the Y-axis (**a**), and a comparison of pH-dependent solubility diagrams for gibbsite (**b**) and goethite (**c**) according to Hiemstra and van Hove (2019) with the shapes of the respective ${\int }_{c}^{(\eta ;{pK}_{a})}$ curves from Eqs. (26–27), and ${\int }_{c}^{(\eta ;{pK}_{a})}$ curves obtained for Al_2_O_3_ and goethite

In the following step, two variants of Eq. (7) (with *FA*_>*3.5C*_ or *FA*_>*2.5L*_) were tested by replacing *Al(PA)* and *H(PA)* with subsequent predictors from Eqs. (8)–(11). The highest R_a_^2^ values were obtained when *Al(PA)* was replaced with (8.2-pH) *FA*_>*3.5C*_ or (8.2-pH) *FA*_>*2.5L*_, and when *H(PA)* was replaced with (8.2-pH) *Al(T)* and (8.2-pH) *Fe(T)* (Eqs. (15) and (23) in Fig. 4a). R_a_^2^ values increased (Eqs. (18) and (26) in Fig. 4a) when the term (8.2-pH) was replaced with Eq. (4) (where ${\mathrm{f}}_{n}$ was denoted as ${\mathrm{f}}_{p}$). One *pK*_*a*_ value was fitted for six PAAHs on the initial assumption *η* = 1. Notably, the *HA* and *HU* variables were also replaced with the terms ${\mathrm{f}}_{p }HA$ and ${\mathrm{f}}_{p}HU$, respectively, but the predicted variance of *K*_*d*_ decreased.

The effect of the changes in coefficient* η* on *K*_*d*_ prediction was examined in the last step. The values of R_a_^2^ increased along with a decrease in *η*. The results obtained for *η* = 0.2 are shown in Fig. 4a (Eqs. (19) and (27)). Higher R_a_^2^ values for *η* <  < 1 indicate that the adsorption of PAAH anions increased rapidly when positively charged sorption sites appeared in soils. This is because the number of active adsorbent sites rapidly exceeded the number of PAAH anions in the soil suspension. The pH values in the lowest parts of the ${\mathrm{f}}_{p}$ curves with *η* = 0.2 for $Al(T)$ and *FA*_>*2.5L*_ (Fig. 4b) indicated that the adsorption of PAAH anions began at pH of < 7.5. According to the gibbsite solubility diagram proposed by Hiemstra and van Hove (2019), at this pH, Al^3+^ cations from partially dissolved gibbsite appear in a solution. Similarly, the pH values associated with the lowest parts of ${\mathrm{f}}_{p}$ curves for *Fe(T)* (Fig. 4c) signalized that the adsorption of anionic forms of PAAHs began at pH of < 5, i.e., in the pH range where Fe^3+^ cations from partially dissolved goethite appeared in a solution (Hiemstra and van Hove 2019).

Figure 4ab indicates that *pK*_*a*_ values for *Al(T)* and *FA*_>*2.5L*_ or *FA*_>*3.5C*_ variables are very similar. When one *pK*_*a*_ value for *Al(T)* and *FA*_>*2.5L*_ or *Al(T)* and *FA*_>*3.5L*_ was applied, the range of the obtained R_a_^2^ values was identical to that noted in Eqs. (19) and (27). Moreover, a comparison of the models containing *FA*_>*3.5C*_ and *FA*_>*2.5L*_ variables (Fig. 4a) revealed that R_a_^2^ values were highly similar, but in the vast majority of cases, they were higher in models with *FA*_>*2.5L*_, which validates the assumption that *FA* has lognormal like distribution in soil pores (Eq. (6)). Therefore, the mechanistic model that describes the adsorption of PAAHs in soils more clearly, and a little more precisely, than Eq. (7) based just on regression analysis is

28 $${K}_{d}={\Phi }_{n}{FA}_{>2.5L} {\kappa }_{{FA}_{>2.5L}.n}+{\Phi }_{an}(HA {\kappa }_{HA.an}+HU {\kappa }_{HU.an}+\left({FA}_{>2.5L} {\kappa }_{{FA}_{>2.5L}.an}+Al\left(T\right) {\kappa }_{Al\left(T\right).an} \right){\mathrm{f}}_{p}^{\left(\eta ,{ pKa}_{Al\left(III\right)}\right)})+Fe\left(T\right) {\mathrm{f}}_{p}^{\left(\eta ,{ pKa}_{Fe\left(T\right)}\right)}{\kappa }_{Fe\left(T\right).an})$$

In Eq. (28), ${pKa}_{Al\left(III\right)}$ is the activity constant that describes pH-dependent changes in the number of sorption sites exhibiting affinity for anionic forms of PAAHs. The above sites are associated with extractable Al^3+^ species adsorbed by *FA*, as well as with the sites of Al oxyhydroxides. The regression coefficients for Eq. (28) and other details are presented in Supplementary Table S11. The plots of observed vs. predicted values produced minimally better results for Eq. (28) than for Eq. (7) (Supplementary Fig. S2). When the normality of the residuals for both models was tested with the Shapiro–Wilk test, the results for Eq. (28) were much better (Supplementary Table S13). As was mentioned earlier, parameters of the Lasso regression models presented in this study were optimized using the default for the R Glmnet package tenfold cross-validation (training set:test set, 90:10). When parameters of Eqs. (7) and (28) were optimized using fivefold cross-validation (training set:test set, 80:20), results for Eq. (7) were almost identical (Supplementary Table S10) to those for tenfold cross-validation. The obtained *R*_*a*_^2^ values were slightly lower for Eq. (28), but none of its predictors were removed during cross-validation (Supplementary Table S11).

It should be noted that the adsorption of PAAHs was studied in soils with a quite wide range of pH 4.2–7.7. In the case when only soils with pH ≤ 5.6 were used, the *FA*_>*2.5L*_ variable was responsible for the adsorption of the neutral forms of PAAHs, whereas only *Al(PA)* and *H(PA)* variables were sufficient for the adsorption of the anionic forms of PAAHs in Eq. (7). In Eq. (28), the *FA*_>*2.5L*_ variable was responsible for the adsorption of the neutral and anionic forms of PAAHs, whereas *Al(T)* and *Fe (T)*${f}_{p}^{(0.1;4.6)}$ variables were sufficient for the adsorption of the neutral forms of PAAHs. In an analysis of soils with pH ≥ 6.6, more accurate results were obtained with the use of Eq. (28), where *HA*, *HU*, and *FA*_>*2.5L*_ ${f}_{p}^{(0.1;7.3)}$ variables were responsible for the adsorption of the anionic forms of PAAHs. The conclusion was that for soils with a narrower pH range, simplified versions of Eqs. (7) and (28) can be used. The results of the analyses involving soils with low and high pH are described in detail in Section S6.

### Adsorption of PAAHs on fractions of humic substances

#### Adsorption on FA

**Fig. 5:**
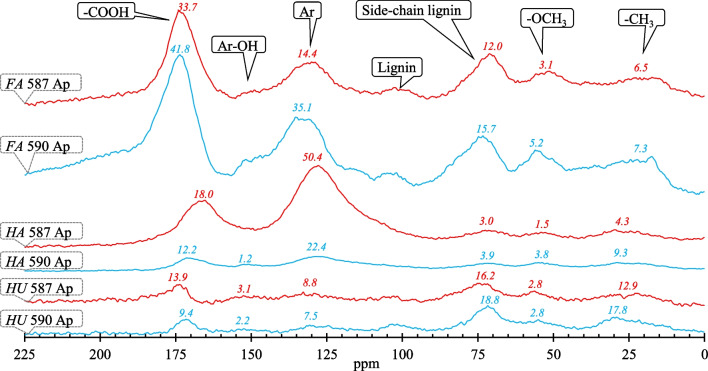
^13^C CP/MAS NMR spectra for humic substance fractions extracted from the Ap horizon of 587 and 590 profiles. Small numbers close to the peaks – integral areas (% of the entire spectrum)

The ^13^C CP/MAS NMR spectra of the fractions, isolated for adsorption experiments to validate Lasso regression results, are presented in Fig. 5. Supplementary Table S2 presents their elemental composition. The obtained spectra were typical (Keeler et al. 2006) and similar for the same types of humic substances, but significant differences were noted across fractions. *FA* fractions were characterized by the largest areas of chemical shift assignments for carboxylic C. The largest areas of aliphatic C bands were observed for *HU*, followed by *FA* fractions. *HA* fractions were characterized by the largest area of aromatic C and the smallest area of aliphatic C bands. Hydrophobicity, calculated based on NMR spectra, increased in the following order: *FA* < *HU* < *HA* (Supplementary Table S14).

**Fig. 6:**
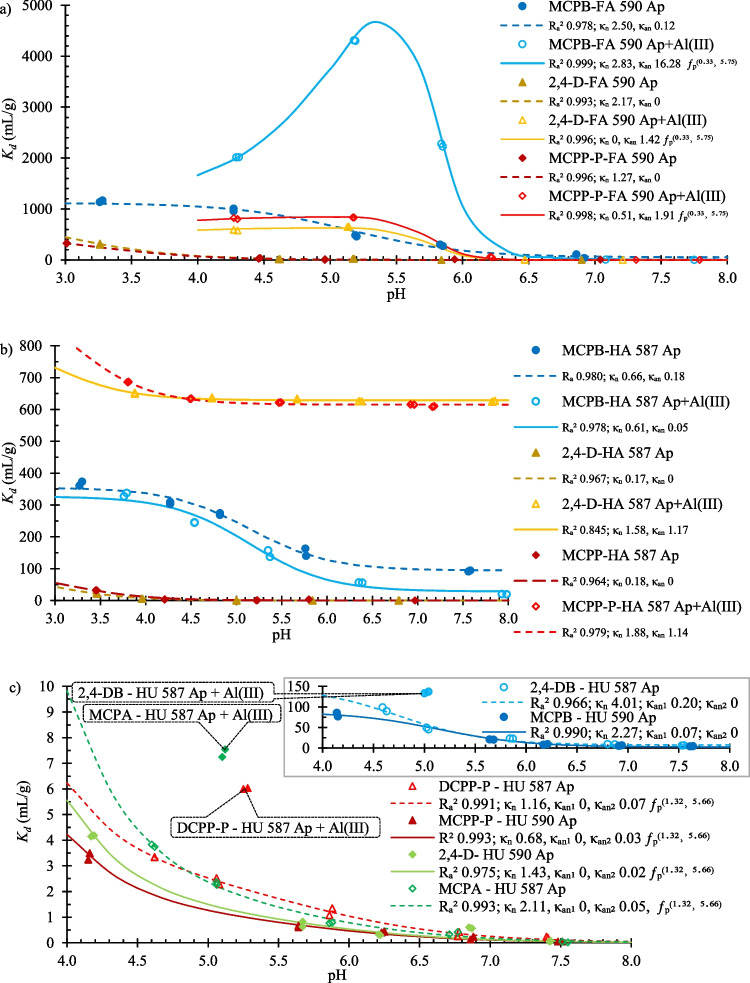
pH-dependent adsorption of PAAHs on selected fractions of humic substances; (**a**) on pure *FA* 590 Ap or after the adsorption of 102.37 mmol( +) Al^3+^/g *OC*; (**b**) on pure *HA* 587 Ap and after the adsorption of 1.61 mmol( +) Al^3+^/g *OC*; (**c**) on pure *HU* 587 Ap and *HU* 590 Ap or on *HU* 587 Ap after the adsorption of 1.74 mmol( +) Al^3+^/g *OC* at pH ~ 5. Used models: ${K}_{d}={\Phi }_{n} X {\kappa }_{n}+{\Phi }_{an} X {\kappa }_{an},$ ${K}_{d}={\Phi }_{n} X {\kappa }_{n}+{\Phi }_{an} X {\kappa }_{an} {\mathrm{f}}_{p}^{\left(\eta , pKa\right)}$ or ${K}_{d}={\Phi }_{n} X {\kappa }_{n}+{\Phi }_{an} X {\kappa }_{an1}+{\Phi }_{an} X {\kappa }_{an2} {\mathrm{f}}_{p}^{\left(\eta , pKa\right)}$, where *X* is *OC* content (mg/g) of *FA*, *HA* or *HU*

Figure 6a indicates that pure *FA* adsorbed neutral forms of PAAHs. Modeling results (including also the data for all PAAHs at pH ~ 2.9 and ~ 5.1 for the two isolated *FA* fractions (Supplementary Table S5)) suggested that the adsorption of the anionic forms of herbicides was not significant in the majority of cases (higher R_a_^2^ values on the assumption that *κ*_*an*_ = 0). The adsorption of PAAHs after the complexation of Al^3+^ species by *FA* was tested in the next step. The addition of 51.18 mmol( +) Al^3+^/g *OC* did not affect adsorption, but adsorption significantly increased (Fig. 6a; refer also to Supplementary Tables S4 and S5) after the addition of 102.37 mmol( +) Al^3+^/g *OC* (i.e., 20 times more than the average amount obtained for *FA*_>*2.5L*_ in Eq. (8)). Such a large amount was necessary because the tested *FA* were completely saturated with H^+^. Therefore, 40.90 (*FA* 590 Ap) to 79.57 (*FA* 587 Ap) mmol Na^+^/g *OC* had to be added to bring their solutions to a pH of ~ 5.4. In addition to the adsorption of the neutral forms of PAAHs, significant adsorption of their anionic forms was also observed in *FA* samples complexed with Al^3+^. The adsorption models revealed that the number of sorption sites associated with Al^3+^ was strongly pH-dependent. The obtained value of *pK*_*a*_ (5.75) and the need to add a large amount of Al^3+^ suggested, that adsorption was associated with extractable Al^3+^ species. Thus, the results of the experiments involving pure *FA* and *FA* with adsorbed Al^3+^ were fully consistent with the results of Lasso regression for *Al(PA)* (Eqs. (8) and (9)) and the results for soils (Eq. (28)).

Wu et al. (2020) examined the adsorption of MCPA on fractions of dissolved organic matter and observed complex interactions (hydrophobic force, ligand exchange and hydrogen bonding) between the herbicide and hydrophobic fraction, largely identified with fulvic acids. Fluorescence studies examining 2,4-D adsorption (Larrivee et al. 2003) and DCPP adsorption (Elkins et al. 2011) demonstrated that anionic forms of the herbicides were bound to *FA* mostly through an Al^3+^ bridge. The present study revealed that the above adsorption mechanism is found for all examined PAAHs.

#### Adsorption on HA

The adsorption of PAAHs on pure *HA* was considerably smaller than the adsorption on *FA* (Fig. 6b). The adsorption models revealed that only neutral forms of 2,4-D and MCPP-P (selected as representatives of acetic acid and propionic acid derivatives, respectively) were adsorbed and neutral and anionic forms of MCPB (representative of butyric acid derivatives). This observation contradicts Eq. (28), which assumes that *HA* primarily adsorbs PAAH anions. Therefore, it was assumed that, similarly to *FA*, adsorption of anionic form of PAAHs was associated with the Al^3+^ species adsorbed by *HA*.

In Eqs. (8) and (9), *HA* was not identified as an adsorbent that significantly contributed to *(Al)PA*. However, the penalty function applied in Lasso regression eliminates the least important predictors. The penalty function applied in Ridge regression generally assumes that all predictors of the regression model are important (Zumel and Mount 2019). The following model was obtained (R_a_^2^ 0.915, λ_optimal_ 0.052) when Ridge regression was used to predict *Al(PA)* using the same predictors as in Eq. (8), as well as the *HA* variable:

29 $$Al\left(PA\right)=\left(8.2-pH\right) \left(0.0008 {Sand}^{\left(0.48\right)}+0.1473 {FA}_{>2.5L}^{\left(0.40\right)}+0.0061 {HA}^{\left(0.06\right)}+ 0.0045 {HU}^{\left(0.18\right)}\right)$$

The lowest value of the standardized regression coefficient for *HA* (0.06) indicated that this was the least important predictor of the model. The value of R_a_^2^ was also lower in Eq. (29) than in Eq. (8). However, when the effect of increasing Al^3+^ adsorption on *HA* on the adsorption of PAAHs was tested, the addition of 1.61 mmol( +) Al^3+^/g *OC* (i.e., 10 times more than the average value for *HA* in Eq. (29)) led to the strong adsorption of 2,4-D and MCPP-P anions and partial inhibition of the adsorption of MCPB anions (Fig. 6b). The ƒ_p_ formula was not useful for describing the adsorption of the anionic forms of PAAHs by *HA* complexed with Al^3+^ species. Notably, Eq. (28) assumes that the adsorption of anionic herbicides on *HA* occurs on pH-independent sorption sites. The obtained results suggest that the adsorption of 2,4-DB and MCPB anions on *HA* was not mediated (or mediated to a very small extent) by the bridges formed by the Al^3+^ species but by direct PAAH–HA bonds. The existence of the pH-independent sorption sites on *HA* complexed with Al^3+^ species suggests, that adsorption of 2,4-D, MCPA, MCPP-P, and DCPP-P anions, in addition to bridging or complexation with Al^3+^, involved other adsorption mechanisms, e.g., it was associated with the high hydrophobicity of *HA* (Supplementary Table S14).

The study on the adsorption of 2,4-D on *HA* at pH 3.3–3.6 was conducted by Khan (1973). The author revealed that adsorption was an endothermic process with a physical character. The adsorption of 2,4-D at pH 2.9 and 4.6 (Celis et al. 1999), MCPA at pH 4.1 (Iglesias et al. 2010), and MCPA and 2,4-D at pH 2.9 (Ćwieląg-Piasecka et al. 2018) followed the same pattern as in Fig. 6b, i.e., the lower the pH, the greater the adsorption. *HA* can interact with Al^3+^ species because they have a high content of carboxyl, hydroxyl, and carbonyl groups (Piccolo and Stevenson 1982; Provenzano et al. 2004) (Fig. 5). Complexes of Al^3+^ with *HA* were examined recently by Jin et al. (2018). To the best of our knowledge, there are no published data on the adsorption of PAAHs on *HA* complexed with Al^3+^.

#### Adsorption on HU

The adsorption of neutral (higher) as well as anionic forms of PAAHs (lower) was observed on the pure *HU* fractions (Fig. 6c). Adsorption modeling revealed that *HU* had two types of sorption sites that could adsorb PAAH anions. The anionic forms of DCPP-P, MCPP-P, 2,4-D and MCPA were predominantly adsorbed on the pH-dependent sorption sites (*κ*_*an2*_ in Fig. 6c), whereas MCPB and 2,4-DB anions were adsorbed on the pH-independent sites (*κ*_*an1*_ in Fig. 6c). The estimated *pK*_*a*_ value of 5.66 suggested that the pH-dependent sorption sites are associated with Al^3+^ species adsorbed on the *HU* surface. Because the Al^3+^ species were also adsorbed by *HU* (Eqs. (8), (9) and (29)), the *HU* samples complexed with Al^3+^ were assessed. A significant increase in the adsorption of anionic forms of PAAHs was observed only after the previous addition of 1.74 mmol( +) Al^3+^/g *OC* (Fig. 6c), i.e., 20 times more than the average amount obtained for *HU* in Eq. (8).

Microscopic analysis (Supplementary Fig. S5 and Table S16) revealed that clusters with a high concentration of *OC* (525 ± 103 mg/g for *HU* 587 Ap and 415 ± 184 mg/g for *HU* 587 Ap, on average) also contained Al (2.01% and 3.51% on average, respectively). However, the *HU* samples still contained mineral soil components that were not dissolved with HF, including illite grains, a mixture of ferric hydroxide minerals (goethite) with silica (quartz) and accessory rutile, and ilmenite (Ce, La, Nd, and Sm) grains. High concentrations of Al (~ 100 mg/g) were observed on illite and chlorite grains, as well as on goethite. Al^3+^ on clay minerals should have a structural character, whereas Al^3+^ on iron oxyhydroxides and humic substances can be interpreted as surface Al^3+^ species. Therefore, it is likely that the pH-dependent sorption sites of the examined *HU* samples (Fig. 6c) were associated with the Al^3+^ species bound with humins and, possibly, with the Al^3+^ species loosely bound on the surface of the mineral soil components. To the best of our knowledge, there are no published data concerning the adsorption of PAAHs on *HU* or regarding adsorption in the ternary *HU*–Al^3+^–PAAH system.

The results of Eq. (28) (Supplementary Table S12) indicate that in the examined soils, 13.4%–62.6% (36.8% on average) of the tested PAAHs were adsorbed by water-soluble low-molecular-weight *FA*, and 14.4%–28.6% of the herbicides were adsorbed by *HA*. The above results suggest that after intensive rainfall, a part of the freshly dissolved *FA* with adsorbed PAAHs (directly or via bridges with Al^3+^) can be transported to groundwater as well as to surface water via runoff. PAAHs are most often quickly degraded in pore water; however, they are weakly available to soil microbiota following adsorption (Ogram et al. 1985; Paszko et al. 2016), and the results of the present study (Eq. (28), Fig. 6) suggest that the transport of PAAHs with dissolved organic matter is a significant mechanism of surface water and groundwater contamination. This mechanism of PAAH transport to surface water and groundwater was suggested recently in studies investigating the adsorption of MCPA in fractions of dissolved organic matter (Wu et al. (2018); Wu et al. (2020). To the best of our knowledge, this is the first study to demonstrate that a large part of PAAHs is adsorbed by water-soluble humic substances in soils.

### Adsorption of PAAHs on Al_2_O_3_ and goethite

**Fig. 7:**
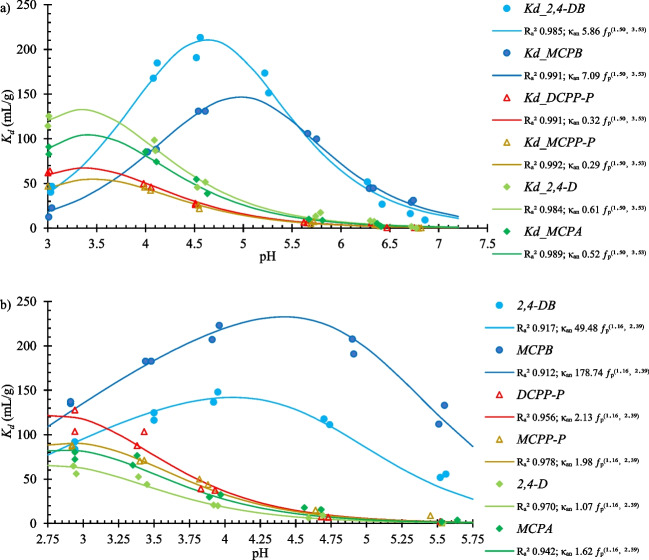
pH-dependent adsorption of PAAHs (**a**) on Al_2_O_3_ and (**b**) goethite. Used model: ${K}_{d}={\Phi }_{an} X {\kappa }_{an} {\mathrm{f}}_{p}^{\left(\eta , pKa\right)}$, where *X* is Al or Fe content (g/kg)

When Al_2_O_3_ was used as the adsorbent (Fig. 7a), only anionic forms of PAAHs were adsorbed, and only pH-dependent sorption sites were observed. The shape of the ƒ_p_ curve (η = 1.50, *pK*_*a*_ = 3.53) indicated that adsorption occurred at pH of < 7 (Fig. 4b). An analysis of PAAH adsorption on goethite (Fig. 7b), which was selected as a representative of Fe oxyhydroxides, produced similar results to those noted for Al_2_O_3_: Only anionic forms of herbicides were adsorbed on pH-dependent sorption sites of this soil mineral. The obtained ƒ_p_ curve (η = 1.16, *pK*_*a*_ = 2.39; Fig. 4c) shows that the sites active toward the anionic forms of PAAHs appeared at pH of < 5.

The results reported by Addorisio et al. (2010) and Sannino et al. (1997) suggested that the adsorption of anionic forms of PAAHs on Al and Fe oxyhydroxides is dominated by electrostatic attraction. By contrast, Kersten et al. (2014) postulated that strong inner-sphere interactions between the goethite surface and neutral MCPA molecules contribute to the adsorption of MCPA on goethite. The results of the present study suggest that anionic forms of PAAHs are adsorbed within a pH range at which terminal > Fe^+^ and/or − FeOH^+^ as well as > Al^+^ and/or − AlOH^+^ sites occur in charged (not hydrolyzed) forms (Fig. 4bc), implying that electrostatic attraction is the dominant adsorption mechanism.

The adsorption of PAAHs, examined on α-alumina at pH 5.6 and 7.0 (Clausen et al. 2001; Sannino et al. 1997) and on goethite, ferrihydrite, and lepidocrocite at acidic pH (Celis et al. 1999; Clausen and Fabricius 2001; Kersten et al. 2014), has been often attributed to the fact that these soil minerals are positively charged. The pH of the point of zero charge (PZC) is 8.0–9.0 for gibbsite and α-alumina and 6.7–9.2 for goethite, ferrihydrite, and lepidocrocite (Clausen and Fabricius 2001; Clausen et al. 2001; Sposito 1989). However, in the cited studies, PAAHs were adsorbed at a pH of at least one unit lower than the PZC of the examined minerals. The PZC of Al_2_O_3_ from IoLiTec applied in the present study is 9.1 (Addorisio et al. 2010), and the PZC of goethite from Sigma-Aldrich is 6.9 (Yu et al. 2019). The *pK*_*a*_ values obtained for the sorption sites of Al_2_O_3_ and goethite (Fig. 4bc) are inconsistent with any of the above values. Kersten et al. (2014) suggested that the adsorption of MCPA on goethite is controlled by aqueous speciation rather than the PZC of the adsorbent. The present results indicate that the amount of terminal Al^3+^ and Fe^3+^ species with unsatisfied valence that can be extracted using moderately effective extraction solutions, such as BaCl_2_–TEA or Tamm’s solution, as well as the *pK*_*a*_ values of these species, associated with their hydrolysis constants, are crucial for describing the adsorption of the anionic form of PAAHs in soils.

## Conclusions

The study revealed that neutral forms of PAAHs were adsorbed by fulvic acids covering soil mesopores with a size of > 2.5 nm, whereas their anionic forms were adsorbed on the pH-dependent sorption sites of this fraction associated with adsorbed Al^3+^ species, which acted as bridges between fulvic acids and PAAHs. Humic acids, humins, and Al and Fe oxyhydroxides adsorbed predominantly anionic forms of PAAHs. In humic substance fractions, PAAH adsorption on humic acids was the most complex process. 2,4-D, MCPA, MCPP-P, and DCPP-P anions were adsorbed by humic acids via bridges formed by Al^3+^ species. However, a different mechanism, associated with, e.g., the high hydrophobicity of this fraction occurred, was responsible for the fact that the sorption sites of *HA* were pH-independent. It appears that 2,4-DB and MCPB anions were adsorbed directly by humic acids. Adsorption experiments on isolated humins revealed two types of sorption sites that could adsorb anionic forms of PAAHs: pH-dependent sites that were most likely associated with adsorbed Al^3+^ and pH-independent sites where anions were directly bound with humins. These observations indicate that Al^3+^ bridging is the key mechanism by which anionic forms of PAAHs are adsorbed on humic substances.

The modeling of PAAH adsorption in soils supported the creation of two models. The simpler model (Eq. (7)) was based on fractions of humic substances and predictors related to the potential acidity of soils. In the more extensive model (Eq. (28)), in addition to humic substance fractions, the contents of Al and Fe oxyhydroxides were used as predictors, and the formula allowing the estimation of the activity range of the pH-dependent sorption sites of soils. These models demonstrated that the amount of the terminal Al^3+^ and Fe^3+^ cations with unsatisfied valence that could be extracted using moderately effective extraction solutions, such as BaCl_2_–TEA or Tamm’s solution, as well as their *pK*_*a*_ values, are crucial for determining the adsorption of the anionic forms of PAAHs in soils. We believe that these models can accurately predict the adsorption of PAAHs not only in Arenosol, Luvisol, and Chernozem profiles in the temperate climate but also in similar soil groups in similar climates.

The observation that PAAHs are adsorbed primarily by fulvic and humic acids in soils was one of the most important findings of this study. This observation confirms recent suggestions that transport with soluble organic matter can be an important mechanism of groundwater and surface water contamination with PAAHs.

## Supplementary Information

Below is the link to the electronic supplementary material.

Supplementary file1 (PDF 1.58 MB)

## Data Availability

All data analyzed and generated in this study are included in this published article and its Supplementary information file.

## References

[CR1] Addorisio V, Esposito S, Sannino F (2010) Sorption capacity of mesoporous metal oxides for the removal of MCPA from polluted waters. J Agr Food Chem 58:5011–5016. 10.1021/jf9044815 20329794 10.1021/jf9044815

[CR2] Albert A, Serjeant EP (1984) The determination of ionization constants. A laboratory manual, 3rd edn. Chapman and Hall, London

[CR3] Audette Y, Longstaffe JG, Gillespie AW, Smith DS, Voroney RP (2021) Validation and comparisons of NaOH and Na_3_PO_4_ extraction methods for the characterization of organic amendments. Soil Sci Soc Am J 85:273–285. 10.1002/saj2.20195

[CR4] Bai Y, Li X, Li Z (2018) Effect of Fe/Al oxides on the measurement of soil exchangeable acidity by BaCl_2_-TEA extraction method. Southwest China J Agr Sci 31:1851–1855. 10.16213/j.cnki.scjas.2018.9.015. (in Chinese)

[CR5] Barrett EP, Joyner LG, Halenda PP (1951) The determination of pore volume and area distributions in porous substances. 1. Computations from nitrogen isotherms. J Am Chem Soc 73:373–380. 10.1021/ja01145a126

[CR6] Bieganowski A, Witkowska-Walczak B, Gliński J, Sokołowska Z, Sławinski C, Brzezinska M, Włodarczyk T (2013) Database of Polish arable mineral soils: A review. Int Agrophys 27:335–350. 10.2478/intag-2013-0003

[CR7] Bogan BW, Sullivan WR, Cruz KH, Paterek JR, Ravikovitch PI, Neimark AV (2003) “Humic coverage index” as a determining factor governing strain-specific hydrocarbon availability to contaminant-degrading bacteria in soils. Environ Sci Technol 37:5168–5174. 10.1021/es030425w 14655703 10.1021/es030425w

[CR8] Cave MR, Harmon K (1997) Determination of trace metal distributions in the iron oxide phases of red bed sandstones by chemometric analysis of whole rock and selective leachate data. Analyst 122:501–512. 10.1039/a607953i

[CR9] Celis R, Hermosin MC, Cox L, Cornejo J (1999) Sorption of 2,4-dichlorophenoxyacetic acid by model particles simulating naturally occurring soil colloids. Environ Sci Technol 33:1200–1206. 10.1021/Es980659t

[CR10] ChemAxon (2024) Marvin - A full featured chemical editor for making science accessible on all platforms. https://chemaxon.com/products/marvin. Accessed 20 Apr 2024

[CR11] Clausen L, Fabricius I (2001) Atrazine, isoproturon, mecoprop, 2,4-D, and bentazone adsorption onto iron oxides. J Environ Qual 30:858–869. 10.2134/jeq2001.303858x 11401274 10.2134/jeq2001.303858x

[CR12] Clausen L, Fabricius I, Madsen L (2001) Adsorption of pesticides onto quartz, calcite, kaolinite, and alpha-alumina. J Environ Qual 30:846–857. 10.2134/jeq2001.303846x 11401273 10.2134/jeq2001.303846x

[CR13] Curtin D, Rostad HPW (1997) Cation exchange and buffer potential of Saskatchewan soils estimated from texture, organic matter and pH. Can J Soil Sci 77:621–626. 10.4141/S97-015

[CR14] Curtin D, Huang PM, Rostad HPW (1987) Components and particle-size distribution of soil titratable acidity. Soil Sci Soc Am J 51:332–336. 10.2136/sssaj1987.03615995005100020013x

[CR15] Ćwieląg-Piasecka I, Medyńska-Juraszek A, Jerzykiewicz M, Dębicka M, Bekier J, Jamroz E, Kawałko D (2018) Humic acid and biochar as specific sorbents of pesticides. J Soils Sediments 18:2692–2702. 10.1007/s11368-018-1976-5

[CR16] De Paolis F, Kukkonen J (1997) Binding of organic pollutants to humic and fulvic acids: Influence of pH and the structure of humic material. Chemosphere 34:1693–1704. 10.1016/S0045-6535(97)00026-X

[CR17] Elkins KM, Dickerson MA, Traudt EM (2011) Fluorescence characterization of the interaction Suwannee river fulvic acid with the herbicide dichlorprop (2-(2,4-dichlorophenoxy)propionic acid) in the absence and presence of aluminum or erbium. J Inorg Biochem 105:1469–1476. 10.1016/j.jinorgbio.2011.08.009 21983257 10.1016/j.jinorgbio.2011.08.009

[CR18] EU Pesticides Database (2024) European Commision. https://ec.europa.eu/food/plants/pesticides/eu-pesticides-database_en. Accessed 24 Apr 2024

[CR19] Fox PM, Nico PS, Tfaily MM, Heckman K, Davis JA (2017) Characterization of natural organic matter in low-carbon sediments: extraction and analytical approaches. Org Geochem 114:12–22. 10.1016/j.orggeochem.2017.08.009

[CR20] Franco A, Trapp S (2008) Estimation of the soil-water partition coefficient normalized to organic carbon for ionizable organic chemicals. Environ Toxicol Chem 27:1995–2004. 10.1897/07-583.1 18384236 10.1897/07-583.1

[CR21] Franco A, Trapp S (2010) A multimedia activity model for jonizable compounds: validation study with 2,4-dichlorophenoxyacetic acid, aniline, and trimethoprim. Environ Toxicol Chem 29:789–799. 10.1002/Etc.115 20821507 10.1002/etc.115

[CR22] Gregor JE, Powell HKJ (1986) Acid pyrophosphate extraction of soil fulvic-acids. J Soil Sci 37:577–585. 10.1111/j.1365-2389.1986.tb00389.x

[CR23] Haberhauer G, Pfeiffer L, Gerzabek MH (2000) Influence of molecular structure on sorption of phenoxyalkanoic herbicides on soil and its particle size fractions. J Agr Food Chem 48:3722–3727. 10.1021/Jf9912856 10956177 10.1021/jf9912856

[CR24] Haberhauer G, Temmel B, Gerzabek MH (2002) Influence of dissolved humic substances on the leaching of MCPA in a soil column experiment. Chemosphere 46:495–499. 10.1016/S0045-6535(01)00194-1 11838426 10.1016/s0045-6535(01)00194-1

[CR25] Hiemstra T, van Hove B (2019) Chemical processes in soil-water-atmosphere: Elements of soil, water and atmospheric chemistry. Wageningen University & Research, Wageningen

[CR26] Iglesias A, López R, Gondar D, Antelo J, Fiol S, Arce F (2009) Effect of pH and ionic strength on the binding of paraquat and MCPA by soil fulvic and humic acids. Chemosphere 76:107–113. 10.1016/j.chemosphere.2009.02.012 19269671 10.1016/j.chemosphere.2009.02.012

[CR27] Iglesias A, López R, Gondar D, Antelo J, Fiol S, Arce F (2010) Adsorption of MCPA on goethite and humic acid-coated goethite. Chemosphere 78:1403–1408. 10.1016/j.chemosphere.2009.12.063 20083293 10.1016/j.chemosphere.2009.12.063

[CR28] IHSS (2024) Isolation of IHSS soil fulvic and humic acids. https://humic-substances.org/isolation-of-ihss-soil-fulvic-and-humic-acids/. Accessed 26 March 2024

[CR29] ISO 11260 (2018) Soil quality - Determination of effective cation exchange capacity and base saturation level using barium chloride solution. https://www.iso.org/standard/60566.html. Accessed 31 Oct 2024

[CR30] ISO 13536 (1995) Soil quality - Determination of the potential cation exchange capacity and exchangeable cations using barium chloride solution buffered at pH = 8.1. https://www.iso.org/standard/22180.html. Accessed 31 Oct 2024

[CR31] ISO 14254 (2018) Soil quality - Determination of exchangeable acidity using barium chloride solution as extractant. https://www.iso.org/standard/60567.html. Accessed 31 Oct 2024

[CR32] Jafvert CT (1990) Sorption of organic acid compounds to sediments: Initial model development. Environ Toxicol Chem 9:1259–1268. 10.1897/1552-8618(1990)9[1259:Sooact]2.0.Co;2

[CR33] Jarvis N (2016) Extended sorption partitioning models for pesticide leaching risk assessments: Can we improve upon the *k*_*oc*_ concept? Sci Total Environ 539:294–303. 10.1016/j.scitotenv.2015.09.002 26363724 10.1016/j.scitotenv.2015.09.002

[CR34] Jarvis N (2018) Meta-analysis of pesticide sorption in subsoil. Environ Toxicol Chem 37:755–761. 10.1002/etc.4011 29057488 10.1002/etc.4011

[CR35] Jin PK, Song JN, Wang XCC, Jin X (2018) Two-dimensional correlation spectroscopic analysis on the interaction between humic acids and aluminum coagulant. J Environ Sci 64:181–189. 10.1016/j.jes.2017.06.018 10.1016/j.jes.2017.06.018 29478638

[CR36] Kah M, Brown CD (2006) Adsorption of ionisable pesticides in soils. Rev Environ Contam T 188:149–217. 10.1007/978-0-387-32964-2_5 10.1007/978-0-387-32964-2_5 17016919

[CR37] Kah M, Brown CD (2007) Prediction of the adsorption of ionizable pesticides in soils. J Agr Food Chem 55:2312–2322. 10.1021/Jf063048q 17295514 10.1021/jf063048q

[CR38] Keeler C, Kelly EF, Maciel GE (2006) Chemical-structural information from solid-state ^13^C NMR studies of a suite of humic materials from a lower montane forest soil, Colorado, USA. Geoderma 130:124–140. 10.1016/j.geoderma.2005.01.015

[CR39] Kersten M, Tunega D, Georgieva I, Vlasova N, Branscheid R (2014) Adsorption of the herbicide 4-chloro-2-methylphenoxyacetic acid (MCPA) by goethite. Environ Sci Technol 48:11803–11810. 10.1021/es502444c 25251872 10.1021/es502444c

[CR40] Khan SU (1973) Equilibrium and kinetic studies of adsorption of 2,4-D and picloram on humic acid. Can J Soil Sci 53:429–434. 10.4141/cjss73-060

[CR41] Kodama H, Schnitzer M (1971) Evidence for interlamellar adsorption of organic matter by clay in a podzol soil. Can J Soil Sci 51:509–512. 10.4141/cjss71-067

[CR42] Larrivee EM, Elkins KM, Andrews SE, Nelson DJ (2003) Fluorescence characterization of the interaction of Al and Pd with Suwannee River fulvic acid in the absence and presence of the herbicide 2,4-dichlorophenoxyacetic acid. J Inorg Biochem 97:32–45. 10.1016/S0162-0134(03)00239-3 14507458 10.1016/s0162-0134(03)00239-3

[CR43] Lewis KA, Tzilivakis J, Warner DJ, Green A (2016) An international database for pesticide risk assessments and management. Hum Ecol Risk Assess 22:1050–1064. 10.1080/10807039.2015.1133242

[CR44] Loos R, Gawlik BM, Locoro G, Rimaviciute E, Contini S, Bidoglio G (2009) EU-wide survey of polar organic persistent pollutants in European river waters. Environ Pollut 157:561–568. 10.1016/j.envpol.2008.09.020 18952330 10.1016/j.envpol.2008.09.020

[CR45] Loos R, Locoro G, Comero S, Contini S, Schwesig D, Werres F, Balsaa P, Gans O, Weiss S, Blaha L, Bolchi M, Gawlik BM (2010) Pan-European survey on the occurrence of selected polar organic persistent pollutants in ground water. Water Res 44:4115–4126. 10.1016/j.watres.2010.05.032 20554303 10.1016/j.watres.2010.05.032

[CR46] Lumivero (2023) XLSTAT - Advanced analytics in Excel. https://lumivero.com/products/xlstat/. Accessed 20 April 202

[CR47] McBride MB (1995) Environmental chemistry of soils. Oxford University Press, New York

[CR48] McLaren RG, Crawford DV (1973) Studies on soil copper. 1. Fractionation of copper in soils. J Soil Sci 24:172–181. 10.1111/j.1365-2389.1973.tb00753.x

[CR49] OECD (2000) OECD guideline for the testing of chemicals. Method 106. Adsorption-desorption using batch equilibrium method. OECD, Paris

[CR50] Ogram AV, Jessup RE, Ou LT, Rao PSC (1985) Effects of sorption on biological degradation rates of (2,4-dichlorophenoxy)acetic acid in soils. Appl Environ Microbiol 49:582–587. 10.1128/aem.49.3.582-587.1985 3994366 10.1128/aem.49.3.582-587.1985 PMC373553

[CR51] Paszko T (2014) Modeling of pH-dependent adsorption and leaching of MCPA in profiles of Polish mineral soils. Sci Total Environ 494–495:229–240. 10.1016/j.scitotenv.2014.06.129 10.1016/j.scitotenv.2014.06.129 25051325

[CR52] Paszko T, Spadotto CA (2022) Modeling of bentazone leaching in soils with low organic matter content. Int J Environ Res Public Health 19:7187. 10.3390/ijerph19127187 35742436 10.3390/ijerph19127187 PMC9223228

[CR53] Paszko T, Muszyński P, Materska M, Bojanowska M, Kostecka M, Jackowska I (2016) Adsorption and degradation of phenoxyalkanoic acid herbicides in soils: A review. Environ Toxicol Chem 35:271–286. 10.1002/etc.3212 26292078 10.1002/etc.3212

[CR54] Paszko T, Matysiak J, Kaminski D, Pasieczna-Patkowska S, Huber M, Krol B (2020) Adsorption of bentazone in the profiles of mineral soils with low organic matter content. PLoS ONE 15:0242980. 10.1371/journal.pone.0242980 10.1371/journal.pone.0242980 PMC7710104 33264340

[CR55] Piccolo A, Stevenson FJ (1982) Infrared-spectra of Cu^2+^, Pb^2+^, and Ca^2+^ complexes of soil humic substances. Geoderma 27:195–208. 10.1016/0016-7061(82)90030-1

[CR56] Prado AGS, Airoldi C (2001) Adsorption and preconcentration of 2,4-dichlorophenoxyacetic acid on a chemically modified silica gel surface. Fresen J Anal Chem 371:1028–1030. 10.1007/s00216-001-1097-6 10.1007/s00216-001-1097-6 11769793

[CR57] Provenzano MR, D’Orazio V, Jerzykiewicz M, Senesi N (2004) Fluorescence behaviour of Zn and Ni complexes of humic acids from different sources. Chemosphere 55:885–892. 10.1016/j.chemosphere.2003.11.040 15041293 10.1016/j.chemosphere.2003.11.040

[CR58] Ravikovitch PI, Bogan BW, Neimark AV (2005) Nitrogen and carbon dioxide adsorption by soils. Environ Sci Technol 39:4990–4995. 10.1021/es048307b 16053101 10.1021/es048307b

[CR59] Rumpel C, Rabia N, Derenne S, Quenea K, Eusterhues K, Kögel-Knabner I, Mariotti A (2006) Alteration of soil organic matter following treatment with hydrofluoric acid (HF). Org Geochem 37:1437–1451. 10.1016/j.orggeochem.2006.07.001

[CR60] Sannino F, Violante A, Gianfreda L (1997) Adsorption-desorption of 2,4-D by hydroxy aluminium montmorillonite complexes. Pestic Sci 51:429–435. 10.1002/(SICI)1096-9063(199712)51:4%3c429::AID-PS619%3e3.0.CO;2-J

[CR61] Siek M, Paszko T (2019) Factors affecting coupled degradation and time-dependent sorption processes of tebuconazole in mineral soil profiles. Sci Total Environ 690:1035–1047. 10.1016/j.scitotenv.2019.06.409 31302536 10.1016/j.scitotenv.2019.06.409

[CR62] Siek M, Paszko T, Jerzykiewicz M, Matysiak J, Wojcieszek U (2021) Mechanisms of tebuconazole adsorption in profiles of mineral soils. Molecules 26:4728. 10.3390/molecules26164728 34443316 10.3390/molecules26164728 PMC8398351

[CR63] Spadotto CA, Hornsby AG (2003) Soil sorption of acidic pesticides: Modeling pH effects. J Environ Qual 32:949–956. 10.2134/jeq2003.9490 12809295 10.2134/jeq2003.9490

[CR64] Sposito G (1989) The chemistry of soils. Oxford Univestity Press, New York

[CR65] Stevenson FJ (1994) Humus chemistry: Genesis, comoposition, reactions. John Willey & Sons, INC., New York

[CR66] Tόth G, Montanarella L, Stolbovoy V, Máté F, Bόdis K, Jones A, Panagos P, Van Liedekerke M (2008) Soils of the European Union. Office for Official Publications of the European Communities, Luxembourg

[CR67] Wada K, Okamura Y (1977) Measurements of exchange capacities and hydrolysis as means of characterizing cation and anion retentions by soils. Proceedings of the International Seminar on Soil Environment and Fertility Management in Intensive Agriculture. International Seminar on Soil Environment and Fertility Management in Intensive Agriculture, Soc. Sci. Soil Manure, Japan, pp 811–815

[CR68] Wavefunction (2011) Spartan'10 for windows, macintosh and linux. Available online: http://downloads.wavefun.com/Spartan10Manual.pdf. Accessed 20 April 202

[CR69] Werner D, Garratt JA, Pigott G (2013) Sorption of 2,4-D and other phenoxy herbicides to soil, organic matter, and minerals. J Soils Sediments 13:129–139. 10.1007/s11368-012-0589-7

[CR70] WRB (2015) WRB, World reference base for soil resources 2014, update 2015. International soil classification system for naming soils and creating legends for soil maps. World soil Resources Reports No. 106. FAO, Rome

[CR71] Wu DM, Yun YH, Jiang L, Wu CY (2018) Influence of dissolved organic matter on sorption and desorption of MCPA in ferralsol. Sci Total Environ 616:1449–1456. 10.1016/j.scitotenv.2017.10.169 29070453 10.1016/j.scitotenv.2017.10.169

[CR72] Wu DM, Ren CQ, Jiang L, Li QF, Zhang W, Wu CY (2020) Characteristic of dissolved organic matter polar fractions with variable sources by spectrum technologies: Chemical properties and interaction with phenoxy herbicide. Sci Total Environ 724:10.1016/j.scitotenv.2020.138262 10.1016/j.scitotenv.2020.138262 32272408

[CR73] Xing BS, Pignatello JJ (1997) Dual-mode sorption of low-polarity compounds in glassy poly(vinyl chloride) and soil organic matter. Environ Sci Technol 31:792–799. 10.1021/es960481f

[CR74] Yu CL, Devlin JF, Bi EP (2019) Bonding of monocarboxylic acids, monophenols and nonpolar compounds onto goethite. Chemosphere 214:158–167. 10.1016/j.chemosphere.2018.09.080 30265922 10.1016/j.chemosphere.2018.09.080

[CR75] Zumel N, Mount J (2019) Practical data science with R. Manning Publications Co., Shelter Island, New York

