# Supplementary material for: Can the pH-dependent adsorption of phenoxyalkanoic herbicides in soils be described with a single equation?
Source: Environ Sci Pollut Res Int. 2024 Nov 8;31(55):63926–45. doi: 10.1007/s11356-024-35413-0 (PMC11602863; doi:10.1007/s11356-024-35413-0)
Supplement: Supplementary file 1 — Supplementary file1 (PDF 1.58 MB) [file 11356_2024_35413_MOESM1_ESM.pdf]

Environmental Science and Pollution Research

Supplementary information for:

Can the pH-dependent adsorption of phenoxyalkanoic herbicides in soils be described with a single equation?

Tadeusz Paszko[a*], Claudio A. Spadotto[b], Miłosz Huber[c], Maria Jerzykiewicz[d], Joanna Matysiak[a], Alicja Skrzypek[a], Patrycja Boguta[e]

[a]Department of Chemistry, University of Life Sciences, Akademicka 15, 20-950 Lublin, Poland;

[b]Embrapa Digital Agriculture, Av. André Tosello, 209, Campinas (SP) 13083-886, Brazil;

[c]Department of Geology, Soil Science and Geoinformation, Maria Curie-Skłodowska University, Kraśnicka 2d/107, 20-718 Lublin, Poland;

[d]Faculty of Chemistry, University of Wrocław, F. Joliot-Curie 4, 50-383 Wrocław, Poland;

[e]Institute of Agrophysics, Polish Academy of Sciences, Doświadczalna 4, Lublin, 20-290, Poland.

[*]Corresponding author.

E-mail address: tadeusz.paszko@up.lublin.pl; Telephone: 00 48 81 445 6640; Fax: 00 48 81 533 3549

S1. Soil properties

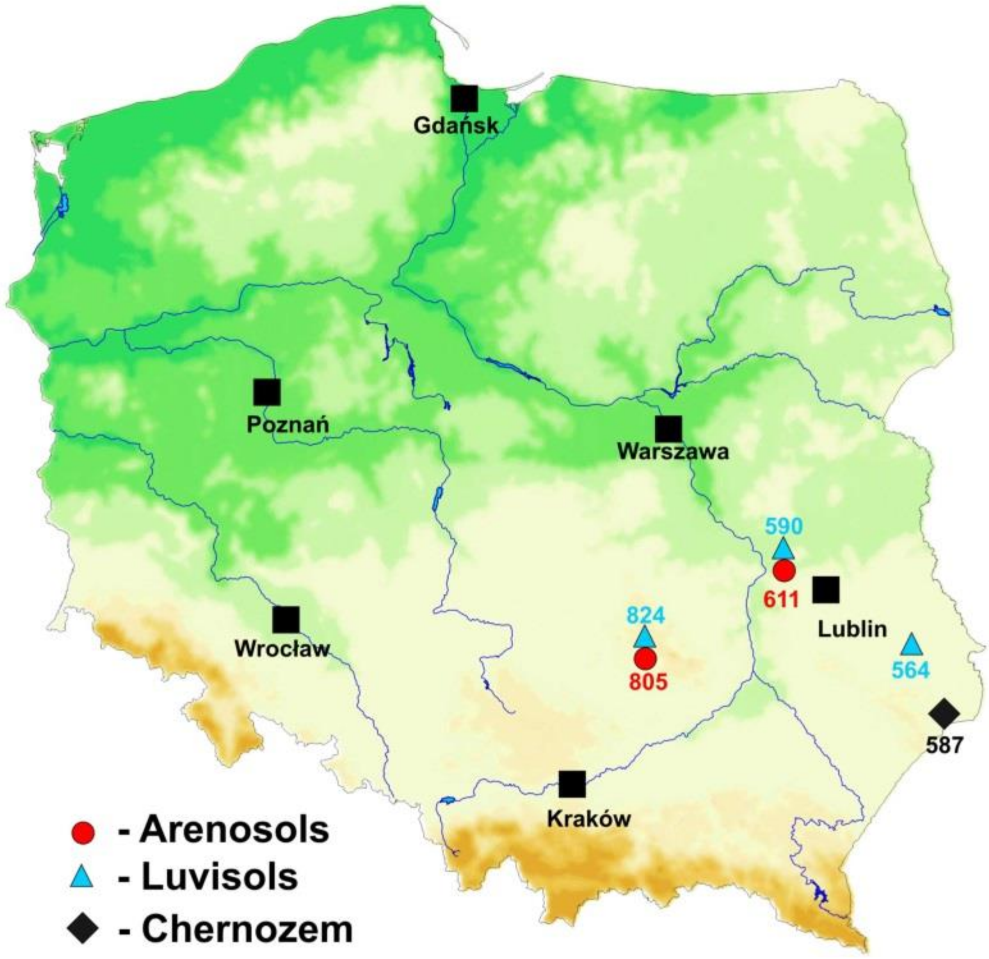

Fig. S1. Locations of examined soil profiles on the map of Poland.

Table S1. Physico-chemical properties of soils.

Location	Olempin			Dęba			Skierbieszów			Ulhówek			Górno			Leszczyny		
(Latitude; Longitude)	(51°23’56’’N; 22°14’14’’E)			(51°26’17’’N; 22°10’14’’E)			(50°50’36’’N; 23°21’57’’E)			(50°27’36’’N; 23°47’35’’E)			(50°51’18’’N; 20°45’38’’E)			(50°52’51’’N; 20°45’48’’E)		
Soil group[a]	Arenosol			Luvisol			Luvisol			Chernozem			Arenosol			Luvisol		
Soil code[b]	611			590			564			587			805			824		
Horizon[a]	Ap	BC	C	Ap	Bt1	Bt2	Ap	Bt1	Bt2	Ap	A2	AC	Ap	BC	C	Ap	Bt1	Bt2
Sand (%)[c]	88.50	96.50	98.00	78.00	57.00	48.00	18.00	16.00	14.00	16.50	15.50	14.50	85.00	95.00	89.50	51.50	62.50	66.50
Silt (%)[c]	9.67	2.42	0.97	17.65	20.00	21.98	72.21	71.52	61.08	70.60	64.58	67.74	12.25	4.10	7.18	39.81	22.13	22.51
Clay (%)[c]	1.83	1.08	1.03	4.35	23.00	30.02	9.79	12.48	24.92	12.90	19.92	17.76	2.75	0.90	3.32	8.69	15.37	10.99
pH[d]	4.96	4.62	4.54	4.45	4.75	4.58	6.97	6.78	6.85	7.08	7.32	7.48	4.20	5.06	5.57	5.46	4.52	4.57
Al(T) (g/kg)[e]	0.51	0.39	0.31	0.58	0.84	0.81	0.68	0.77	1.22	0.88	1.25	0.92	0.40	0.17	0.23	0.85	0.71	0.62
Fe(T) (g/kg)[e]	1.43	0.49	0.40	2.44	5.78	4.81	3.53	3.57	4.35	2.58	3.83	3.71	2.15	0.38	0.94	4.56	3.86	5.53
OC (mg/g)[f]	11.303	0.896	0.339	8.822	1.588	0.878	13.546	4.783	3.131	19.210	13.138	6.661	6.807	0.424	0.415	10.755	1.896	1.090
FA (mg/g)[g]	1.640	0.330	0.261	1.800	0.548	0.301	1.547	0.702	0.604	1.491	1.188	1.081	1.378	0.242	0.251	1.770	0.687	0.488
HA (mg/g)[g]	1.759	0.179	0.009	1.712	0.321	0.289	1.508	1.110	0.266	3.973	5.660	2.093	1.110	0.149	0.155	1.404	0.436	0.213
HU (mg/g)[g]	7.904	0.387	0.068	5.310	0.719	0.287	10.491	2.972	2.260	13.746	6.290	3.487	4.320	0.033	0.009	7.581	0.774	0.389
ECEC (cmol(+)/kg)[h]	1.528	0.760	0.784	2.155	8.469	10.825	9.587	8.215	12.212	12.429	12.196	11.735	1.346	0.746	1.531	5.394	5.919	4.677
EA (cmol(+)/kg)[i]	0.124	0.216	0.256	0.356	0.182	0.225	0.005	0	0	0.005	0	0	0.469	0.07	0.004	0.054	0.477	0.287
Al(EA) (cmol(+)/kg)[j]	0.036	0.161	0.191	0.107	0.044	0.075	0.000	0	0	0	0	0	0.211	0.006	0.000	0.000	0.228	0.139
H(EA) (cmol(+)/kg[i]	0.088	0.055	0.065	0.249	0.138	0.151	0.005	0	0	0.005	0	0	0.258	0.064	0.004	0.054	0.249	0.148
PCEC (cmol(+)/kg)[k]	4.763	1.252	1.144	5.628	11.359	12.660	12.249	10.269	15.597	16.128	15.799	14.370	3.715	0.699	1.794	9.784	9.266	7.218
PA (cmol(+)/kg)[l]	5.891	2.388	1.921	6.048	7.765	9.048	3.295	3.387	5.011	2.696	2.887	1.419	5.795	1.417	1.73	9.229	7.865	6.419
Al(PA) (cmol(+)/kg)[i]	0.698	0.406	0.295	0.558	0.158	0.135	0.168	0.048	0.019	0.035	0.058	0.025	0.619	0.192	0.161	0.396	0.357	0.338
H(PA) (cmol(+)/kg)[l]	5.194	1.983	1.625	5.489	7.607	8.913	3.127	3.339	4.992	2.661	2.828	1.394	5.176	1.225	1.569	8.833	7.509	6.081
AEC (cmol(-)/kg)[m]	0.076	0.042	0.113	0.156	0.223	0.155	0.056	0.066	0.037	0.071	0.017	0.094	0.118	0.086	0.094	0.090	0.189	0.129
$SSA(aN_2)$ (m^2/g)[n]	0.40	1.28	1.34	2.32	20.04	27.86	6.89	11.25	29.61	14.05	17.05	17.87	1.02	0.77	2.64	7.34	14.48	12.54

SBJH(dN_2) (m^2/g)[o]	0.41	0.75	0.79	1.89	15.97	22.34	6.90	9.23	23.10	9.29	13.77	12.95	0.83	0.56	2.05	5.99	10.41	9.25
$r_{(dN2)mean}$ (nm)[o]	7.045	4.392	3.815	4.926	3.425	3.336	3.281	3.155	2.859	3.03	2.888	3.362	6.151	4.059	3.608	4.411	3.242	3.448
$r_{(dN2)<1.5\ nm}$ (%)[o]	0	0	0	0	0.287	0.162	0.078	0.609	0.299	0.424	0.388	0.503	0	0	0	0.026	0.704	0.716
$r_{(dN2)1.5-1.8\ nm}$ (%)[o]	4.402	9.416	8.524	8.187	11.546	11.075	6.415	11.018	11.680	8.991	8.792	10.609	6.192	8.249	11.874	10.937	13.315	13.038
$r_{(dN2)1.8-2.0\ nm}$ (%)[o]	18.149	31.487	37.601	33.742	34.648	37.191	45.207	41.428	36.757	49.427	50.592	44.798	24.627	40.510	39.816	33.644	38.867	35.088
$r_{(dN2)2.0-2.5\ nm}$ (%)[o]	13.783	14.224	16.858	15.728	18.190	18.688	22.177	18.575	21.988	20.563	20.309	18.216	13.911	15.855	16.645	16.032	18.671	17.088
$r_{(dN2)2.5-3.5\ nm}$ (%)[o]	12.706	13.047	12.148	10.533	14.133	13.191	9.219	12.019	15.451	7.110	7.830	9.025	11.883	10.417	11.016	11.460	11.095	12.900
$r_{(dN2)3.5-5.0\ nm}$ (%)[o]	12.760	11.591	9.941	9.066	9.678	9.326	6.684	7.604	7.669	5.445	5.559	6.974	10.919	8.843	7.638	9.164	7.539	9.463
$r_{(dN2)5.0-10.0\ nm}$ (%)[o]	19.350	13.120	9.814	12.021	7.749	7.132	6.316	5.691	4.509	4.895	3.973	5.667	16.249	10.053	7.990	10.578	6.078	7.688
$r_{(dN2)10.0-30.0\ nm}$ (%)[o]	18.850	7.115	5.114	10.723	3.769	3.234	3.904	3.055	1.647	3.145	2.556	4.209	16.219	6.073	5.020	8.159	3.732	4.020

[a] according to WRB (2015); [b] according to Bieganowski et al. (2013); [c] determined using the pipette method (ISO 11277 2020); [d] determined in 0.01 M $CaCl_2$; [e] extracted with Tamm's reagent (Cave and Harmon 1997) and analyzed using a Varian AA280FS Atomic Absorption Spectrometer; [f] determined using a SSM-5000A solid sample module of Shimadzu TCSH analyzer; [g] determined after extraction with 0.1 M sodium pyrophosphate (Fox et al. 2017); [h] extracted with 0.0025 M $BaCl_2$ (ISO 11260 2018); [i] determined by the potentiometric titration with 0.005 M NaOH to pH of 7.8 (ISO 14254 2018); [j] determined with a Varian Carry 60 UV-Vis Spectrophotometer – $\lambda = 550$ nm, eriochrome cyanine R, pH 5.5 (Shokrollahi et al. 2008); [k] determined after extraction with a solution of 0.5 M $BaCl_2$ and 0.17 M triethanolamine ($BaCl_2$-TEA), pH 8.2 (Curtin and Rostad 1997; ISO 13536 1995); [l] determined by the potentiometric titration with 0.1 M HCl to pH of 5.2 (Curtin and Rostad 1997; ISO 13536 1995); [m] determined according to Wada and Okamura (1977); [n] determined from the Brunauer-Emmett-Teller (BET) equation based on N_2 adsorption at 77 K; [o] determined from N_2 desorption isotherms using the Barrett, Joyner and Halenda method (Barrett et al. 1951); $r_{(dN2)i\ nm}$ (%) =100 $SBJH_i$/SBJH.

Table S2. Results of the elemental analysis of the separated soil fractions.

Fraction	C (mg/g)[a]	H (mg/g)[a]	N (mg/g)[a]	S (mg/g)[a]
FA 587 Ap	246.63	29.44	20.57	3.63
FA 590 Ap	447.20	46.50	32.50	6.09
HA 587 Ap	539.43	31.90	31.53	2.59
HA 590 Ap	517.73	46.54	43.90	4.11
HU 587 Ap	37.57	6.61	4.00	0.38
HU 590 Ap	38.83	5.99	3.00	0.19

[a] determined using a Vario El cube CHNS elemental analyzer.

S2. Methods of isolation of humic substance fractions, selected soil analyses, and adsorption experiments

S2.1. Isolation of FA, HA and HU fractions

The samples from the Ap horizon of profiles 587 and 590 were used to isolate *FA*, *HA* and *HU* fractions. *FA* and *HA* were isolated with 0.1 M $Na_4P_2O_7$ (Audette et al. 2021; Gregor and Powell 1986; IHSS 2024), and *HU* fractions were concentrated with 2% HF (Rumpel et al. 2006). Soil samples (100 g) were adjusted to pH ~ 1.5 with 1 M HCl, and the volume of the solution was adjusted with 0.1 M HCl to a solution/solid ratio of 10:1. The samples were agitated at room temperature (~ 22°C) with a rotator (1.5 h, 100 rpm), centrifuged (20 min, 4000 g, 20°C – settings for all centrifugations), and the supernatant (FA_{HCl}) was stored (5°C) for *FA* isolation. Next, ultrapure water (0.05 µS/cm; 10:1) was added three times to the residues; the samples were agitated (20 min, 45 rpm) and centrifuged. Soil residues were neutralized (0.3 M NaOH); 0.1 M $Na_4P_2O_7$ was added (10:1) three times under an atmosphere of N_2; the suspensions were agitated (16 h, 25 rpm) and centrifuged. The collected supernatants ($FA{+}HA_{Na4P2O7}$) were combined and retained for *FA* and *HA* separation. Next, ultrapure water was added three times to the residues, and the samples were agitated (20 min, 45 rpm) and centrifuged each time. In the last step, 2% HF was added to the soil residues (10:1), and the suspensions were agitated (24 h, 25 rpm) and centrifuged. Agitation, followed by centrifugation, was repeated three times when new portions of 2% HF were added, and four-times when ultrapure water was added (30 min, 45 rpm). The obtained *HU* fractions (denoted as *HU* 587 Ap and *HU* 590 Ap) were freeze-dried to remove water.

$FA{+}HA_{Na4P2O7}$ solutions were slowly acidified with 6 M HCl to pH ~ 1.2 with constant stirring. The solutions were left to stand for 16 h, and *FA* supernatants were separated from *HA* sediments by centrifugation. The collected

$FA_{Na4P2O7}$ supernatants were stored (5°C) for the isolation of the *FA* fractions. *HA* sediments were purified by strictly following the IHSS (2024) procedure. The obtained freeze-dried samples were denoted as *HA* 587 Ap and *HA* 590 Ap.

FA were isolated with Amberlite™ XAD7HP (Gregor and Powell 1986) and Amberlite™ IRC120 H resins from Supelco®. In the first step, the resins were cleaned by sequential washing with acetone, hexane, methanol, and ultrapure water (for details, see Audette, Longstaffe, Gillespie, Smith and Voroney (2021)), and were packed into glass columns (30.0 x 2.0 cm) with a fine PVC mesh at the top and the bottom. The outlet of the column was connected to a flow cell with a glass electrode, next to a peristaltic pump. The flow cell was inserted into the PF-12^{Plus} photometer and connected to a pH meter to monitor the absorbance (λ = 345 nm was used) and pH of column leakage. XAD7 and IRC120 columns were saturated with H^+ by passing 0.1 M or 1.0 M HCl at a constant rate of 2.0 mL/min to obtain a steady pH of ~ 1.0 and ~ 0, respectively. Ultrapure water was then passed through the columns until the effluent samples tested positive for Cl^- in the presence of $AgNO_3$.

Next, FA_{HCl} and $FA_{Na4P2O7}$ solutions were combined and passed through the XAD7 column. The column was monitored to ensure that absorbance in the column effluent did not exceed 0.08. Ultrapure water was then passed through the column. When effluent absorbance exceeded 0.1, the peristaltic pump was stopped, the bottom of the XAD column was connected with the top of the IRC120 column, and the flow cell and the peristaltic pump were attached to the bottom of the IRC120 column. Due to the unique properties of IRC120 resin, Na^+ was completely removed from the passing solution, and *FA* were not adsorbed on its surface. The peristaltic pump was then restarted. After passing in total one pore volume of ultrapure water, the 0.1 M NaOH was passed through the columns. The concentrated *FA* solution was collected when two conditions were met: the effluent tested negative for Cl^- anions, and absorbance in the effluent increased to 0.1. The flow of NaOH solution was stopped when absorbance decreased to 0.08 while pH was still below 3.0. The obtained solutions of *FA 587* Ap and *FA 590* Ap were partly freeze-dried, and some of the liquid was stored (5°C) and later used for adsorption experiments.

S2.2. NMR analysis

The ^{13}C NMR spectra of the isolated humic substances were obtained by CP/MAS 13C-NMR spectroscopy. The analysis was performed at 300 MHz in a Bruker AVANCE III NMR device, equipped with a 4-mm narrow MAS probe and operating in a resonance sequence of 13C at 75.45 MHz. Equal portions of humic substances were placed in a zirconium dioxide rotor with Kel-F caps, with a rotation frequency of 10 ± 1 kHz. The spectra were obtained by

collecting 4096 data points from the same number of scans (40 k) with an acquisition time of 49 ms and with a 4-ms recycle delay. Induction-free decays were transformed by applying a zero-filling equal to 4 k and then a 70- Hz line-broadening adjustment. The spectra were processed using Bruker Topspin 4.1.1. software. Integrals were calculated (as % of total area) for selected ranges of each spectrum. Hydrophobicity was calculated as the ratio of ^{13}C hydrocarbon regions (0-45 ppm (CH_3-R) and 110-160 ppm regions (aromatic C)) and hydrophilic regions (45-60 ppm (O-CH_3) and 145-190 ppm regions (phenolic and carboxylic C)) ((Xu et al. 2017); Table S13).

S2.3. Microscopic analysis

The surface of sand grains was examined under a Hitachi SU6600 scanning electron microscope with an EDS attachment (Thermo). Phase maps were generated under low vacuum (10-15 Pa), with a working BSE attachment at 15 kV gun power, and 90 s per point. The surface of *HU* was examined under a Quanta 3D FEG scanning electron microscope with an EDX Octane Elect Plus attachment. The samples were tested under high vacuum conditions (10-3Pa) after gold sputtering.

S2.4. Adsorption in soils, on goethite and Al_2O_3

The batch adsorption experiments were carried out according to the OECD Guideline 106 (OECD 2000) including control and blank samples. The used soil:solution ratio was 1:5 for 2,4-DB and MCPB, and 1:1.5 for 2,4-D, MCPA, DCPP-P and MCPP-P. Triplicate 1 g (2,4-DB, MCPB) or 2 g (2,4-D, MCPA, DCPP-P and MCPP-P) samples of air-dried soil were weighed out into polypropylene test tubes; 0.01 M $CaCl_2$ and $5 \cdot 10^{-5}$ M $HgCl_2$ (biocide) solution was pipetted (3 mL – 2,4-DB, MCPB or 2 mL − 2,4-D, MCPA, DCPP-P and MCPP-P), and the samples were equilibrated overnight. Next, 2 mL of 2,4-DB or MCPB solutions (7.5 mg/L in 0.01 M $CaCl_2$) or 1 mL of 2,4-D, MCPA, DCPP-P or MCPP-P solutions (9.0 mg/L in 0.01 M $CaCl_2$) were pipetted into the respective test tubes. The initial concentration of each PAAH was 3.0 mg/L. The tubes were agitated for 24 h on a rotator (20 rpm; 20± 0.5°C), because in previous batch experiments analyzing the adsorption kinetics of the PAAHs, equilibrium was most often reached within 4–12 h (Matallo et al. 1998; Paszko 2011; Piwowarczyk and Holden 2013; Thorstensen et al. 2001). At the end of agitation, the pH of soil suspensions was measured at 20 ± 0.5°C with a glass electrode, the tubes were centrifuged (20 min, 3300 g, 20 ± 1°C), and the liquid phase was sampled for HPLC analysis. Adsorption was calculated from the difference between the initial concentration and the concentration after 24 h.

Duplicate 15 mg samples of goethite or Al_2O_3 were weighed into polypropylene test tubes. Solutions with variable NaOH content in 0.01 M $CaCl_2$, and with $5 \cdot 10^{-5}$ M $HgCl_2$, were added (6 mL; the goal was to achieve five final pH values, from ~ 2.9 to ~ 5.5 for goethite, and from ~ 3 to ~ 7 for Al_2O_3). The samples were equilibrated overnight; 8 mg/L of PAAH solutions in 0.01 M $CaCl_2$ was pipetted (4 mL; initial concentration of 3.6 mg/L), and the tubes were agitated for 24 h on the rotator (20 rpm; 20± 0.5°C). At the end of agitation, the pH of soil suspensions was measured at 20 ± 0.5°C with a glass electrode, the tubes were centrifuged (20 min, 3300 g, 20 ± 1°C), and the liquid phase was sampled for HPLC analysis.

S2.5. Adsorption on isolated HU fractions.

Duplicate 50 mg samples of *HU* 587 Ap and *HU* 590 Ap were weighed into the polypropylene test tubes. Solutions with variable NaOH content in 0.01 M $CaCl_2$, and with $5 \cdot 10^{-5}$ M $HgCl_2$, were added (1.48 mL; the goal was to achieve five pH values, from ~ 4.6 to ~ 7.5). The samples were equilibrated overnight; 5 mg/L of PAAH solutions in 0.01 M $CaCl_2$ was pipetted (0.36 mL; initial concentration of 1.0 mg/L). The following steps were identical to those described in the adsorption experiments for soils.

The extent to which the pH-dependent adsorption of PAAHs on *HU* fractions was altered by adding Al^{3+} species was also examined. $AlCl_3$ solutions were pipetted (0.5 mL) into the duplicate 50 mg samples of *HU* at concentrations that would lead to the adsorption of 0.09, 0.35, 0.87 or 1.74 mmol(+) Al^{3+}/g *OC* (on the assumption that Al^{3+} would be adsorbed in 100%). The amount of 0.09 mmol(+) Al^{3+}/g *OC* is the average amount obtained from the Eq. (8) for 18 soils, and subsequent values denoted 4-fold, 10-fold and 20-fold multiples of that amount. Next, *HU* suspensions containing $AlCl_3$ were agitated on the rotator (2 h, 20 rpm; 20± 0.5°C). Solutions with variable NaOH content in 0.015 M $CaCl_2$, and with $7.55 \cdot 10^{-5}$ M $HgCl_2$, were added (0.98 mL; the goal was to achieve final pH values ~ 5.0-6.0), and the test tubes were agitated on the rotator (12 h, 20 rpm; 20± 0.5°C). Next, selected PAAH solutions in 0.01 M $CaCl_2$ were pipetted (0.36 mL; initial concentration of 1.0 mg/L). The following steps were identical to those described in the adsorption experiments on pure *HU*.

S2.6. Adsorption on isolated FA and HA fractions.

The adsorption experiments were also carried out using *FA* suspensions with adsorbed Al^{3+} species. Initially, the selected *FA* solution was pipetted into volumetric flasks; the flasks were placed on a magnetic stirrer, and specific volumes of $AlCl_3$ solution were added to induce the adsorption of up to 5.12, 20.47, 51.18 or 102.37 mmol(+) Al^{3+}/g *OC* (arithmetic mean for 18 soils obtained from Eq. (8) for the $FA_{>2.5L}$ variable, and its 4-fold, 10-fold and 20-fold multiples, respectively). Using the magnetic stirrer, pH of the suspensions was slowly adjusted to five values (from ~ 4 to ~ 7.5) with 0.3 M NaOH, and the suspension in each flask was diluted with ultrapure water (the content of *FA* in *FA* 587 Ap and *FA* 590 Ap suspensions was 0.654 and 0.468 mg/mL). *FA* suspensions were vigorously stirred, and portions (2 mL) were pipetted into the membranes. The membrane was closed with a second clip and placed inside a test tube. Finally, test tubes were placed in the rotator and slowly agitated (2 rpm, 120 h, 20 ± 0.5°C). After agitation, membranes were removed from test tubes, the pH of the solutions from test tubes was measured, the tubes were centrifuged (20 min, 3300 g, 20 ± 1°C), and the liquid phase was sampled for HPLC analysis. At each of the five pH values, duplicate samples of three selected PAAHs and one of the tested *FA* (with the lowest content of Al^{3+}, which caused the significant increase of herbicide adsorption) were used in this series of adsorption experiments. The adsorption of duplicate samples of all PAAHs in *FA* 587 Ap and *FA* 590 Ap suspensions (with the same content of Al^{3+}) was analyzed at pH ~ 5.1.

The experiments analyzing the adsorption of PAAHs on *HA* followed a similar protocol to that described in the *FA* analysis. Samples of *HA* were weighed into volumetric flasks, ultrapure water was added, the flasks were placed on a magnetic stirrer, the pH of the suspensions was slowly adjusted to five values (from ~ 3 to ~ 7.5) with 0.1 M NaOH, and the suspension in each flask was diluted to the same volume with ultrapure water (the content of *HA* in each flask was 4.0 mg/mL). The suspensions were vigorously stirred, and 2 mL was pipetted into the membrane. The membrane was closed with second clips and placed inside a test tube. Next, 0.025 M $CaCl_2$ solution with 0.0125% NaN_3 was pipetted (2 mL) into duplicate test tubes, and 5 mg/L of the PAAH solution was added (1 mL; apparent initial concentration of 1 mg/mL). Finally, test tubes were placed in the rotator and slowly agitated (2 rpm, 120 h, 20 ± 0.5°C). After agitation, membranes were removed from test tubes, the pH of the solutions from test tubes was measured, the tubes were centrifuged (20 min, 3300 g, 20 ± 1°C), and the liquid phase was sampled for HPLC analysis.

In the following series of adsorption experiments, *HA* suspensions complexed with Al^{3+} were used. Samples of *HA* of were placed in volumetric flasks, ultrapure water was added, the flasks were placed on a magnetic stirrer, and specified amounts of $AlCl_3$ solution were added to induce the adsorption of up to 0.16, 0.64, 1.61 or 3.21 mmol(+) Al^{3+}/g *OC*. The lowest values were obtained for the *HA* variable from Eqs. (29) or (30), respectively, and subsequent values are the 4-fold, 10-fold and 20-fold multiples of that value. While stirring the suspensions, the pH in the flasks was slowly adjusted to five values, from ~ 3 to ~ 7.5, with 0.1 M NaOH, and the suspension in each flask was diluted to the same volume with

ultrapure water. *HA* suspensions were vigorously stirred, and duplicate portions (2 mL) were pipetted into membranes. The tubing was closed with second clips and placed inside test tubes. The following steps were identical to those described in the experiments of pure *HA* samples.

S2.7. HPLC measurements

The 40 μL aliquots of herbicide solutions were injected into a Waters HPLC device (Waters Corp., Milford, MA, USA) equipped with a Waters 600 quaternary pump and Waters 600 Controller with the Empower 2 software, the Waters In-Line Degasser AF, the Waters 2998 Photodiode Array Detector (DAD), and s Waters 2707 Autosampler. The Thermo Scientific BDS Hypersil C18 column (250 x 4.6 mm, 5 μm particle size) was maintained at 35 ± 0.5°C using the Varian PCB 150 Water Peltier System. 2,4-DB and MCPB were detected using the mobile phases of 65% and 35% acetonitrile (28:72 v/v) in 10 mM chloroacetic buffer (pH 2.4) at a flow rate of 1.6 mL/min and a runtime of 9 min per sample. 2,4-D, MCPA, DCPP-P and MCPP-P were detected using the mobile phases of 35% and 5% acetonitrile (50:50 v/v) in 10 mM acetic buffer (pH 5.3) at a flow rate of 1.5 mL/min and a runtime of 8 min per sample. The detection wavelength was 230 nm. All samples were measured in triplicate. The detection limit was 0.05 mg/L, and the relative standard deviation of variability of results was < 1%.

S3. Adsorption results

Table S3. Results of experiments on the effect of $5 \cdot 10^{-5}$ M $CaCl_2$ on adsorption of DCPP-P. Adsorption of 3 mg/L DCPP-P in 0.01 M $CaCl_2$, soil:solution 1:1.5, duplicate samples, equilibration time 12 h, temp. 23 °C.

	$C_{HgCl_2} = 0$		$C_{HgCl_2} = 5 \cdot 10^{-5}$ M	
Soil	K_d (mL/g)	pH	K_d (mL/g)	pH
611 C	0.084±0.006 [a]	4.65±0.03 [a]	0.089±0.003	4.67±0.06
805 C	0.049±0.009	5.36±0.04	0.043±0.013	5.41±0.01
564 Bt2	0.154±0.001	6.64±0.01	0.154±0.016	6.62±0.05

[a] standard deviation

Table S4. Values of K_d (mL/g) and pH determined for soils from batch experiments.

Soil code	Horizon	2,4-DB						DCPP-P						2,4-D						MCPB						MCPP-P						MCPA					
		K_d			pH			K_d			pH			K_d			pH			K_d			pH			K_d			pH			K_d			pH		
611	Ap	11.01	10.66	10.73	4.92	4.94	4.93	0.51	0.54	0.50	4.78	4.75	4.86	0.51	0.61	0.60	4.83	4.85	4.83	9.29	9.33	9.24	4.95	4.95	4.89	0.41	0.31	0.39	4.82	5.05	4.85	0.45	0.55	0.37	5.00	4.77	5.19
	BC	1.80	2.20	1.93	4.67	4.68	4.67	0.13	0.14	0.13	4.59	4.58	4.58	0.16	0.15	0.16	4.64	4.66	4.65	2.38	1.85	1.73	4.63	4.64	4.63	0.09	0.10	0.09	4.62	4.60	4.59	0.13	0.10	0.11	4.64	4.63	4.63
	C	1.08	1.46	1.00	4.66	4.66	4.65	0.08	0.08	0.09	4.58	4.59	4.62	0.07	0.06	0.08	4.67	4.69	4.69	1.13	1.28	1.36	4.65	4.65	4.67	0.07	0.05	0.04	4.62	4.62	4.64	0.06	0.07	0.07	4.59	4.60	4.55
590	Ap	7.88	7.92	7.99	4.57	4.60	4.56	0.51	0.50	0.53	4.44	4.42	4.40	0.54	0.53	0.56	4.46	4.47	4.46	7.21	6.64	6.75	4.54	4.59	4.54	0.46	0.45	0.45	4.41	4.44	4.45	0.56	0.52	0.56	4.42	4.49	4.45
	Bt1	2.07	1.94	1.67	4.86	4.87	4.88	0.15	0.13	0.14	4.73	4.76	4.77	0.19	0.19	0.19	4.79	4.78	4.80	1.32	1.95	1.51	5.00	4.89	4.90	0.10	0.11	0.10	4.74	4.74	4.75	0.18	0.19	0.19	4.73	4.75	4.75
	Bt2	1.87	1.95	1.87	4.73	4.73	4.72	0.18	0.17	0.17	4.59	4.59	4.56	0.24	0.26	0.25	4.59	4.60	4.61	1.38	1.75	2.03	4.73	4.75	4.74	0.13	0.13	0.13	4.57	4.59	4.60	0.24	0.24	0.23	4.57	4.56	4.54
564	Ap	1.36	1.22	1.39	6.85	6.83	6.84	0.21	0.22	0.22	6.79	6.80	6.81	0.35	0.36	0.36	6.93	6.94	6.91	0.86	0.89	0.77	6.81	6.85	6.85	0.11	0.12	0.12	6.81	6.79	6.82	0.25	0.25	0.24	6.91	6.88	6.89
	Bt1	0.86	1.02	0.80	6.82	6.84	6.83	0.16	0.15	0.16	6.70	6.69	6.74	0.30	0.29	0.29	6.72	6.73	6.72	0.48	0.66	0.55	6.78	6.87	6.83	0.09	0.09	0.09	6.69	6.68	6.70	0.23	0.22	0.21	6.75	6.72	6.76
	Bt2	0.35	0.68	0.43	6.83	6.89	6.84	0.08	0.07	0.07	6.73	6.81	6.82	0.12	0.12	0.12	6.83	6.83	6.86	0.34	0.44	0.39	6.81	6.88	6.88	0.03	0.03	0.02	6.81	6.81	6.81	0.11	0.09	0.09	6.81	6.84	6.86
587	Ap	1.89	1.81	1.84	7.16	7.19	7.21	0.32	0.32	0.32	7.17	7.21	7.21	0.65	0.66	0.66	7.27	7.23	7.23	1.19	1.17	1.19	7.14	7.11	7.12	0.19	0.20	0.20	7.24	7.24	7.23	0.48	0.48	0.48	7.17	7.15	7.16
	A2	1.76	1.71	1.62	7.47	7.46	7.48	0.28	0.28	0.31	7.41	7.44	7.44	0.61	0.67	0.62	7.51	7.52	7.52	0.89	0.88	0.89	7.45	7.46	7.53	0.17	0.19	0.18	7.48	7.48	7.48	0.44	0.48	0.44	7.44	7.49	7.44
	AC	0.56	0.44	0.71	7.62	7.62	7.60	0.13	0.12	0.13	7.58	7.55	7.60	0.24	0.26	0.26	7.70	7.70	7.71	0.41	0.24	0.37	7.61	7.63	7.65	0.07	0.06	0.07	7.63	7.64	7.63	0.17	0.17	0.17	7.63	7.64	7.63
805	Ap	9.80	10.43	10.17	4.38	4.37	4.36	0.65	0.63	0.64	4.27	4.27	4.25	0.68	0.68	0.69	4.35	4.32	4.30	7.33	7.10	7.38	4.48	4.63	4.45	0.57	0.58	0.57	4.32	4.28	4.28	0.64	0.68	0.64	4.27	4.24	4.23
	BC	0.69	1.09	1.13	5.18	5.24	5.25	0.05	0.05	0.05	5.21	5.19	5.22	0.03	0.03	0.03	5.15	5.18	5.21	0.39	0.78	0.71	5.30	5.26	5.26	0.02	0.02	0.02	5.17	5.19	5.17	0.02	0.01	0.02	5.16	5.16	5.17
	C	0.58	0.64	0.68	5.40	5.40	5.45	0.04	0.05	0.05	5.39	5.37	5.38	0.03	0.03	0.03	5.39	5.38	5.41	0.55	0.58	0.59	5.44	5.45	5.47	0.01	0.01	0.01	5.51	5.43	5.41	0.01	0.01	0.02	5.35	5.34	5.35
824	Ap	4.97	5.00	4.50	5.63	5.60	5.56	0.32	0.33	0.32	5.53	5.51	5.53	0.46	0.48	0.42	5.56	5.57	5.56	3.51	3.36	3.48	5.59	5.63	5.61	0.25	0.25	0.24	5.52	5.49	5.53	0.41	0.41	0.42	5.46	5.46	5.46
	Bt1	3.25	3.12	3.25	4.61	4.59	4.59	0.28	0.25	0.24	4.48	4.48	4.48	0.32	0.33	0.33	4.47	4.49	4.50	2.83	3.01	2.63	4.60	4.61	4.62	0.21	0.21	0.21	4.53	4.52	4.50	0.29	0.31	0.31	4.47	4.50	4.47
	Bt2	2.67	2.84	2.69	4.68	4.68	4.68	0.18	0.17	0.17	4.61	4.60	4.60	0.23	0.24	0.22	4.59	4.61	4.61	2.28	2.02	1.95	4.67	4.73	4.69	0.14	0.14	0.14	4.60	4.60	4.59	0.21	0.22	0.21	4.56	4.57	4.60

Table S5. Values of K_d (mL/g) for *FA* 587 Ap and *FA* 590 Ap at pH ~ 2.9 and ~ 5.1.

Fraction code	2,4-DB				DCPP-P				2,4-D				MCPB				MCPP-P				MCPA			
	K_d (SE)		pH		K_d (SE)		pH		K_d (SE)		pH		K_d (SE)		pH		K_d (SE)		pH		K_d (SE)		pH	
FA 587 Ap	1436.0 (17.2)	739.9 (5.8)	2.96	5.07	334.5 (3.6)	94.7 (1.5)	2.93	5.33	285.4 (3.3)	16.7 (5.8)	2.99	5.01	1934.6 (36.2)	666.0 (7.8)	3.04	4.94	370.2 (9.3)	113.0 (4.6)	2.95	5.01	360.3 (3.1)	87.7 (0.3)	2.97	4.98
FA 590 Ap	1839.7 (13.8)	348.5 (4.4)	2.88	5.14	484.1 (10.9)	79.1 (2.3)	2.90	5.51	408.1 (4.9)	13.5 (2.4)	2.90	5.18	2963.9 (47.0)	609.0 (24.8)	2.84	5.20	498.8 (6.1)	125.8 (0.8)	2.91	5.18	532.5 (5.7)	158.5 (1.3)	2.88	5.16

Table S6. Values of K_d (mL/g) at pH ~ 5.4 for *FA* 587 Ap and *FA* 590 Ap after addition of 102.37 mmol(+) Al^{3+}/g *OC*.

Fraction code	2,4-DB		DCPP-P		2,4-D		MCPB		MCPP-P		MCPA	
	K_d (SE)	pH	K_d (SE)	pH	K_d (SE)	pH	K_d (SE)	pH	K_d (SE)	pH	K_d (SE)	pH
FA 587 Ap	1602.28 (49.39)	5.33	441.93 (5.51)	5.57	316.58 (2.11)	5.39	802.47 (21.74)	5.24	461.67 (2.59)	5.33	372.42 (2.02)	5.35
FA 590 Ap	4902.32 (59.33)	5.38	856.05 (15.47)	5.35	619.97 (2.83)	5.40	4178.01 (11.45)	5.38	851.80 (3.83)	5.51	721.11 (50.35)	5.43

S4. Modeling results for soils

Table S7. Results of the Lasso regression modeling of PAAHs adsorption in 18 topsoils and subsoils (n = 54; 10-fold cross-validation).

Eq.	PAAH	Independent variables Φ_n	Φ_{an}	R_a^2	Eq.	PAAH	Independent variables Φ_n	Φ_{an}	R_a^2
(S1)	2,4-DB	*OC*	*OC*	0.891	(S19)	MCPB	*OC*	*OC*	0.912
(S2)	DCPP-P	*OC*	*OC*	0.797	(S20)	MCPP-P	*OC*	*OC*	0.859
(S3)	2,4-D	*OC*	*OC*	0.737	(S21)	MCPA	*OC*	*OC*	0.748
(S4)	2,4-DB	*FA*, *HU*	*FA*	0.910	(S22)	MCPB	*FA*, *HU*	*FA*, *HA*	0.928
(S5)	DCPP-P	*FA*, *HU*	*FA*, *HA*, *HU*	0.889	(S23)	MCPP-P	*FA*, *HU*	*FA*, *HA*	0.914
(S6)	2,4-D	*FA*	*FA*, *HA*, *HU*	0.867	(S24)	MCPA	*FA*	*FA*, *HA*, *HU*	0.874
(S7)	2,4-DB	*OC*, *Sand*	*OC*, *Al(PA)*, *H(PA)*	0.949	(S25)	MCPB	*OC*, *Sand*	*OC*, *Al(PA)*, *H(PA)*, *Al(T)*	0.959
(S8)	DCPP-P	*OC*, *Sand*	*OC*, *Al(PA)*, *H(PA)*, *Al(T)*	0.961	(S26)	MCPP-P	*OC*, *Sand*	*OC*, *Al(PA)*, *H(PA)*	0.959
(S9)	2,4-D	*OC*, *Sand*	*OC*, *H(PA)*, *Fe(T)*	0.896	(S27)	MCPA	*OC*, *Sand*,	*OC*, *H(PA)*, *Al(T)*	0.926
(S10)	2,4-DB	*HU*, *Sand*	*HA*, *HU*, *Al(PA)*, *H(PA)*	0.963	(S28)	MCPB	*FA*, *HU*, *Sand*	*HA*, *HU*, *H(PA)*	0.969
(S11)	DCPP-P	*HU*, *Sand*	*HA*, *HU*, *Al(PA)*, *H(PA)*	0.977	(S29)	MCPP-P	*FA*, *HU*, *Sand*	*HA*, *HU*, *Al(PA)*, *H(PA)*	0.969
(S12)	2,4-D	*HU*, *Sand*	*HA*, *HU*, *H(PA)*	0.958	(S30)	MCPA	*FA*, *HU*, *Sand*	*HA*, *HU*, *Al(PA)*, *H(PA)*	0.967
(S13)	2,4-DB	$FA_{>3.5C}$	*HA*, *HU*, *Al(PA)*, *H(PA)*	0.977	(S31)	MCPB	$FA_{>3.5C}$	*HA*, *Al(PA)*, *H(PA)*	0.970
(S14)	DCPP-P	$FA_{>3.5C}$	*HA*, *HU*, *Al(PA)*, *H(PA)*	0.985	(S32)	MCPP-P	$FA_{>3.5C}$	*HA*, *HU*, *Al(PA)*, *H(PA)*	0.978
(S15)	2,4-D	$FA_{>3.5C}$	*HA*, *HU*, *Al(PA)*, *H(PA)*	0.960	(S33)	MCPA	$FA_{>3.5C}$	*HA*, *HU*, *Al(PA)*, *H(PA)*	0.969
(S16)	2,4-DB	$FA_{>2.5L}$	*HA*, *HU*, *Al(PA)*, *H(PA)*	0.984	(S34)	MCPB	$FA_{>2.5L}$	*HA*, *HU*, *Al(PA)*, *H(PA)*	0.982
(S17)	DCPP-P	$FA_{>2.5L}$	*HA*, *HU*, *Al(PA)*, *H(PA)*	0.987	(S35)	MCPP-P	$FA_{>2.5L}$	*HA*, *HU*, *Al(PA)*, *H(PA)*	0.980
(S18)	2,4-D	$FA_{>2.5L}$	*HA*, *HU*, *Al(PA)*, *H(PA)*	0.963	(S36)	MCPA	$FA_{>2.5L}$	*HA*, *HU*, *Al(PA)*, *H(PA)*	0.970

Table S8. *FA* contents in soil mesopores (mg/g) assuming constant (C) and lognormal like (L) distributions.

Soil group	Arenosol			Luvisol			Luvisol			Chernozem			Arenosol			Luvisol		
Soil code	611			590			564			587			805			824		
Horizon	Ap	BC	C	Ap	Bt1	Bt2	Ap	Bt1	Bt2	Ap	A2	AC	Ap	BC	C	Ap	Bt1	Bt2
$FA_{<1.8C}$[a]	0.072	0.031	0.022	0.147	0.065	0.034	0.100	0.082	0.072	0.140	0.109	0.120	0.085	0.020	0.030	0.194	0.096	0.067
$FA_{<1.8\text{-}2.0C}$[a]	0.298	0.104	0.098	0.608	0.190	0.112	0.699	0.291	0.222	0.737	0.601	0.484	0.339	0.098	0.100	0.595	0.267	0.171
$FA_{2.0\text{-}2.5C}$[a]	0.226	0.047	0.044	0.283	0.100	0.056	0.343	0.130	0.133	0.307	0.241	0.197	0.192	0.038	0.042	0.284	0.128	0.083
$FA_{2.5\text{-}3.5C}$[a]	0.208	0.043	0.032	0.190	0.077	0.040	0.143	0.084	0.093	0.106	0.093	0.098	0.164	0.025	0.028	0.203	0.076	0.063
$FA_{3.5\text{-}5.0C}$[a]	0.209	0.038	0.026	0.163	0.053	0.028	0.103	0.053	0.046	0.081	0.066	0.075	0.150	0.021	0.019	0.162	0.052	0.046
$FA_{5.0\text{-}10.0C}$[a]	0.317	0.043	0.026	0.216	0.042	0.021	0.098	0.040	0.027	0.073	0.047	0.061	0.224	0.024	0.020	0.187	0.042	0.038
$FA_{10.0\text{-}30.0C}$[a]	0.309	0.023	0.013	0.193	0.021	0.010	0.060	0.021	0.010	0.047	0.030	0.046	0.223	0.015	0.013	0.144	0.026	0.020
$FA_{<1.8L}$[b]	0.140	0.047	0.031	0.223	0.089	0.046	0.132	0.106	0.094	0.176	0.135	0.155	0.149	0.028	0.040	0.282	0.126	0.091

$FA_{1.8\text{-}2.0L}$[b]	0.542	0.147	0.130	0.862	0.244	0.141	0.858	0.354	0.269	0.864	0.697	0.586	0.556	0.129	0.126	0.810	0.327	0.218
$FA_{2.0\text{-}2.5L}$[b]	0.351	0.057	0.050	0.342	0.109	0.061	0.359	0.135	0.137	0.307	0.239	0.203	0.267	0.043	0.045	0.329	0.134	0.090
$FA_{2.5\text{-}3.5L}$[b]	0.240	0.039	0.026	0.170	0.063	0.032	0.111	0.065	0.072	0.079	0.068	0.075	0.169	0.021	0.022	0.174	0.059	0.051
$FA_{3.5\text{-}5.0L}$[b]	0.163	0.023	0.015	0.099	0.029	0.015	0.054	0.028	0.024	0.041	0.033	0.039	0.106	0.012	0.010	0.095	0.027	0.025
$FA_{5.0\text{-}30.0L}$[b]	0.203	0.017	0.009	0.103	0.014	0.007	0.035	0.013	0.008	0.025	0.016	0.023	0.130	0.009	0.007	0.080	0.015	0.013

[a] determined with the equation $FA_{x\text{-}yC} = FA\ r_{(dN2)x\text{-}y\ \mathrm{nm}}/100$ and using the respective FA and $r_{(dN2)x\text{-}y\ \mathrm{nm}}$ values from Table S1; [b] determined by fitting the equation $S_{r_i} = S_{min} + \frac{S_{max}}{2^{(\frac{r_i}{r_{0.5}})^2}}$ (S_{min} and S_{max} are the minimum and maximum contents of FA in mesopores, S_{r_i} (mg/m^2) is the FA content in mesopores with radius $r_i = r_{(dN2)x\text{-}y\ \mathrm{nm}}/100$, and $r_{0.5}$ is the radius of mesopores in which the FA content is equal to 0.5 ($S_{max}- S_{min}$)) for the respective FA and $r_{(dN2)x\text{-}y\ \mathrm{nm}}$ values from Table S1, assuming S_{min}/S_{max} = 6 and $r_{0.5}$ = 3.5 nm, by minimizing the term $(FA - \sum_{i=1}^{n} S_{r_i})^2$.

Table S9. Values of the Lasso regression coefficients (n = 54; 10-fold cross-validation) obtained for the model:

$$K_d = \Phi_n\ FA_{>3.5C}\ \kappa_{FA_{>3.5C}.n} + \Phi_{an}\ (HA\ \kappa_{HA.an} + HU\ \kappa_{HU.an} + Al(PA)\ \kappa_{Al(PA).an} + H(PA)\ \kappa_{H(PA).an})$$

	$\kappa_{FA_{>3.5C}.n}$	$\kappa_{HA.an}$	$\kappa_{HU.an}$	$\kappa_{Al(PA).an}$	$\kappa_{H(PA).an}$	R_a^2	Optimal λ
2,4-DB	20.652	0.158	0.020	5.833	0.129	0.977	0.025
DCPP-P	12.596	0.032	0.011	0.198	0.012	0.985	0.002
2,4-D	13.760	0.085	0.016	0.090	0.020	0.960	0.003
MCPB	14.413	0.080	0.000	4.194	0.098	0.970	0.022
MCPP-P	10.384	0.020	0.006	0.120	0.009	0.978	0.001
MCPA	11.202	0.058	0.011	0.057	0.021	0.969	0.002

Table S10. Values of the Lasso regression coefficients obtained for the model

$$K_d = \Phi_n\ FA_{>2.5L}\ \kappa_{FA_{>2.5L}.n} + \Phi_{an}\ (HA\ \kappa_{HA.an} + HU\ \kappa_{HU.an} + Al(PA)\ \kappa_{Al(PA).an} + H(PA)\ \kappa_{H(PA).an})$$

	$\kappa_{FA_{>2.5L}.n}$	$\kappa_{HA.an}$	$\kappa_{HU.an}$	$\kappa_{Al(PA).an}$	$\kappa_{H(PA).an}$	R_a^2	Optimal λ
			10-fold cross-validation				
2,4-DB	31.198	0.152	0.037	4.959	0.110	0.984	0.025
DCPP-P	19.284	0.032	0.011	0.183	0.012	0.987	0.002
2,4-D	21.374	0.085	0.016	0.073	0.020	0.963	0.002
MCPB	21.462	0.086	0.009	3.153	0.085	0.982	0.020
MCPP-P	15.874	0.020	0.006	0.107	0.009	0.980	0.001
MCPA	17.008	0.058	0.012	0.047	0.020	0.970	0.002
			5-fold cross-validation				
2,4-DB	31.198	0.152	0.037	4.959	0.110	0.984	0.025
DCPP-P	19.284	0.032	0.011	0.183	0.012	0.987	0.002
2,4-D	21.374	0.085	0.016	0.073	0.020	0.963	0.002
MCPB	21.462	0.086	0.009	3.153	0.085	0.982	0.020
MCPP-P	15.855	0.020	0.006	0.107	0.009	0.980	0.001
MCPA	17.008	0.058	0.012	0.047	0.020	0.970	0.002

Table S11. Values of the Lasso regression coefficients obtained for Eq. (28), i.e.:

$$K_d = \Phi_n FA_{>2.5L}\ \kappa_{FA_{>2.5L}.n} + \Phi_{an}\big(HA\ \kappa_{HA.an} + HU\ \kappa_{HU.an} + \big(FA_{>2.5L}\ \kappa_{FA_{>2.5L}.an} + Al(T)\ \kappa_{Al(T).an}\big)\mathfrak{f}_p^{(\eta=0.287,\ pKa_{Al(III)}=6.734)}\big) + Fe(T)\ \mathfrak{f}_p^{(\eta=0.287,\ pKa_{Fe(T)}=4.294)}\kappa_{Fe(T).an}\big)$$

	$\kappa_{FA_{>2.5L}.n}$	$\kappa_{HA.an}$	$\kappa_{HU.an}$	$\kappa_{FA_{>2.5L}.an}$	$\kappa_{Al(T).an}$	$\kappa_{Fe(T).an}$	R_a^2	Optimal λ
				10-fold cross-validation				
2,4-DB	25.372	0.270	0.032	10.456	0.283	6.983	0.992	0.023
DCPP-P	12.223	0.040	0.011	0.324	0.088	0.124	0.987	0.002
2,4-D	12.453	0.090	0.021	0.115	0.149	0.180	0.974	0.002
MCPB	20.217	0.126	0.032	4.131	0.636	9.807	0.984	0.019
MCPP-P	9.568	0.026	0.006	0.278	0.042	0.145	0.988	0.001
MCPA	10.986	0.064	0.015	0.123	0.153	0.127	0.979	0.002
				5-fold cross-validation				
2,4-DB	26.480	0.254	0.044	9.176	0.744	3.078	0.986	0.021
DCPP-P	14.181	0.039	0.011	0.289	0.093	0.058	0.972	0.002
2,4-D	16.108	0.089	0.021	0.049	0.168	0.072	0.959	0.002
MCPB	20.794	0.122	0.035	2.898	1.046	4.021	0.981	0.019
MCPP-P	9.925	0.025	0.006	0.268	0.042	0.076	0.963	0.001
MCPA	12.266	0.063	0.015	0.101	0.152	0.064	0.964	0.002

Table S12. Contribution (%) of the predictors from Eq. (28) (see Table S10) to the predicted K_d values for 18 soils (n = 54).

	$FA_{>2.5L.n}$	$HA_{.an}$	$HU_{.an}$	$FA_{>2.5L.an}$	$Al(T)_{.an}$	$Fe(T)_{.an}$
				2,4-DB		
Q1	2.7	1.6	0.3	14.7	0.8	0.0
Mean	34.7	19.3	6.2	28.0	4.2	7.6
Q3	57.6	27.6	11.5	43.0	6.1	10.1
				DCPP-P		
Q1	0.2	7.1	2.4	11.0	8.8	0.0
Mean	11.6	21.5	15.5	18.4	24.3	8.7
Q3	21.9	27.6	24.0	25.5	41.4	14.5
				2,4-D		
Q1	0.1	13.2	3.5	2.6	9.0	0.0
Mean	8.3	28.6	18.6	5.2	30.7	8.7
Q3	16.1	36.2	28.8	8.4	50.8	14.6
				MCPB		
Q1	8.4	0.5	0.2	4.0	1.4	0.0
Mean	49.5	14.4	9.2	11.1	9.6	6.2
Q3	83.8	19.4	16.6	22.0	11.8	8.1
				MCPP-P		
Q1	0.3	5.8	1.5	14.3	5.2	0.0
Mean	15.5	21.3	13.7	21.9	16.3	11.4
Q3	26.6	29.3	21.4	29.6	26.6	18.1
				MCPA		
Q1	0.1	9.7	2.6	2.8	8.9	0.0
Mean	10.8	25.6	16.5	5.9	33.2	7.9
Q3	16.1	34.7	27.4	8.6	53.3	11.8

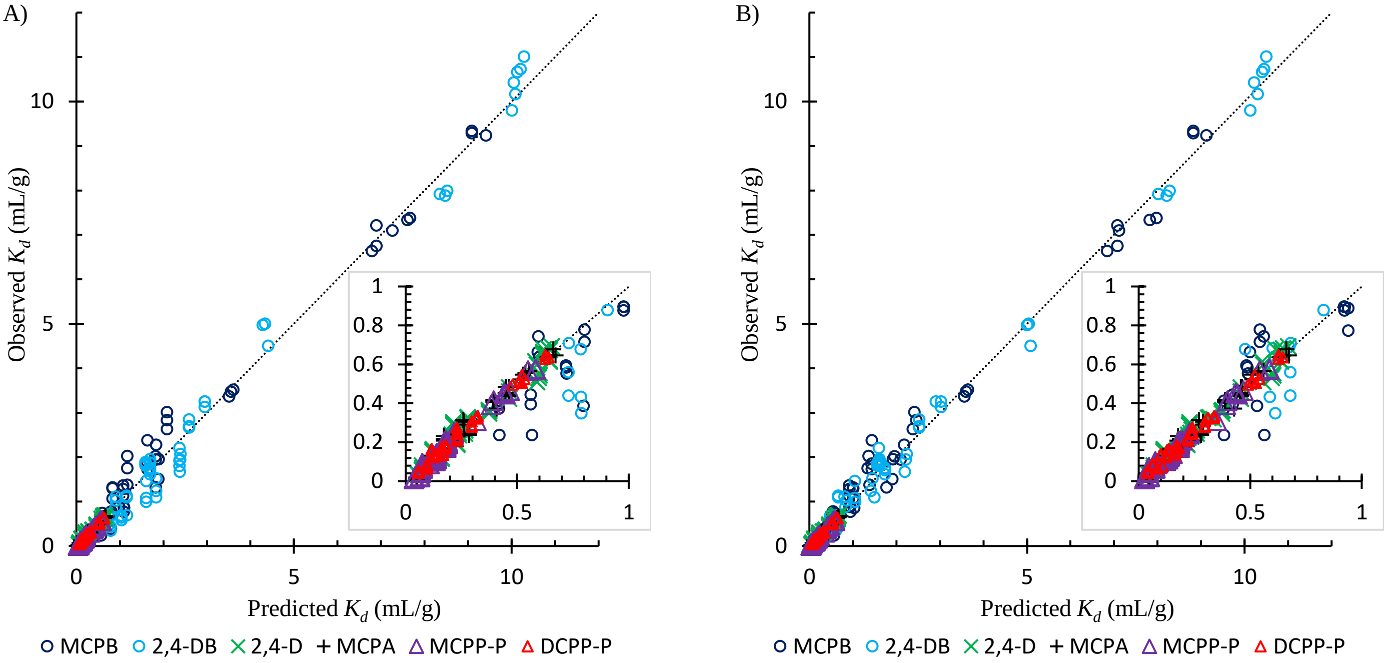

Fig. S2. Observed versus predicted K_d values for (A) Eq. (7) with the $FA_{>2.5L}$ variable (Table S9) and (B) Eq. (28) (Table S10).

Table S13. Results of the normality analysis of residuals for Eq. (7) with the $FA_{>2.5L}$ variable (Table S9) and for Eq. (28) (Table S10) using the Shapiro-Wilk test.

	Eq. (7)		Eq. (28)	
	W	*p*	W	*p*
2,4-DB	0.969	0.172	0.986	0.758
DCPP-P	0.948	0.021	0.983	0.643
2,4-D	0.973	0.253	0.969	0.183
MCPB	0.936	0.006	0.987	0.803
MCPP-P	0.975	0.314	0.984	0.674
MCPA	0.954	0.038	0.974	0.301

S5. Results of instrumental analyses

Table S14. Integrals (%) of selected chemical shifts for ^{13}C CP/MAS NMR spectra of the fractions of humic substances.

	Aliphatic	Ar	CH_3	OCH_3	side-chain lignin	Ar-OH	-COOH	HB[a]
	0-110	110-157	0-43	45-60	43-87	145-165	158-190	
	ppm							
FA 587 Ap	23.0	14.4	6.5	3.1	12.0	nd[b]	33.7	0.57
FA 590 Ap	30.2	35.1	7.3	5.2	15.7	nd	41.8	0.90
HA 587 Ap	8.7	50.4	4.3	1.5	3.0	nd	18.0	2.81
HA 590 Ap	17.0	22.4	9.3	3.8	3.9	1.2	12.2	1.84
HU 587 Ap	34.3	8.8	12.9	2.8	16.2	3.1	13.9	1.10
HU 590 Ap	43.7	7.5	17.8	2.8	18.8	2.2	9.4	1.76

[a] hydrophobicity, determined with: HB = [(0-43) + (110-160)]/[(45-60) + (145-190)] (Xu, Zhao, Chu, Mao, Olk, Xin and Zhang 2017);

[b]nd – not detected, peak too small.

A) B)

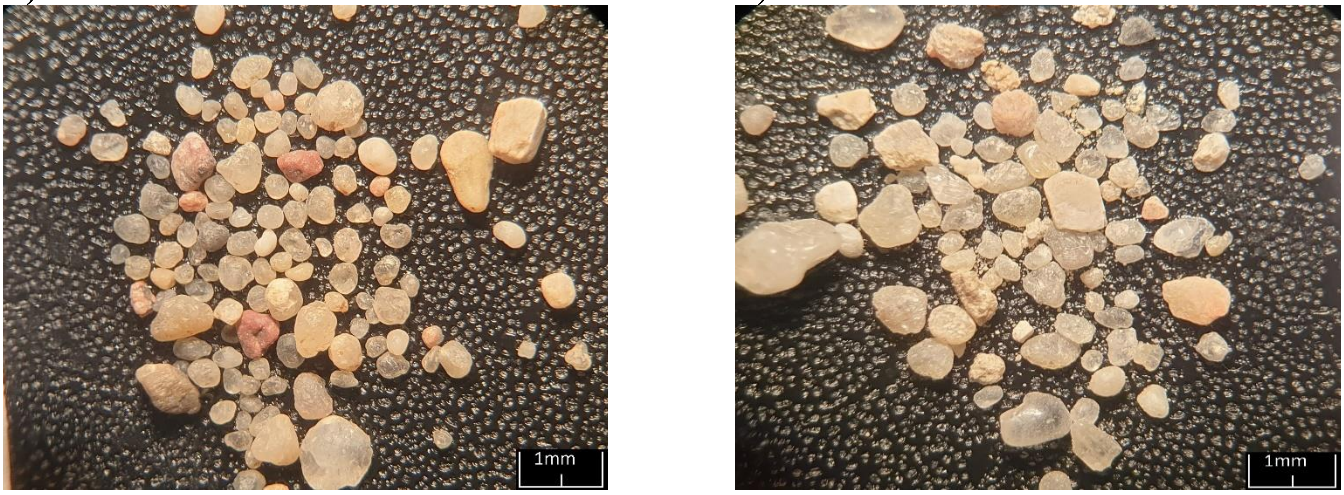

Fig. S3. Optical microscope image (Leica DM2500P) of sand grains from C horizon of (A) 611 and (B) 805 profiles of Arenosols showing the nature of the grains and the degree of rounding.

A) B)

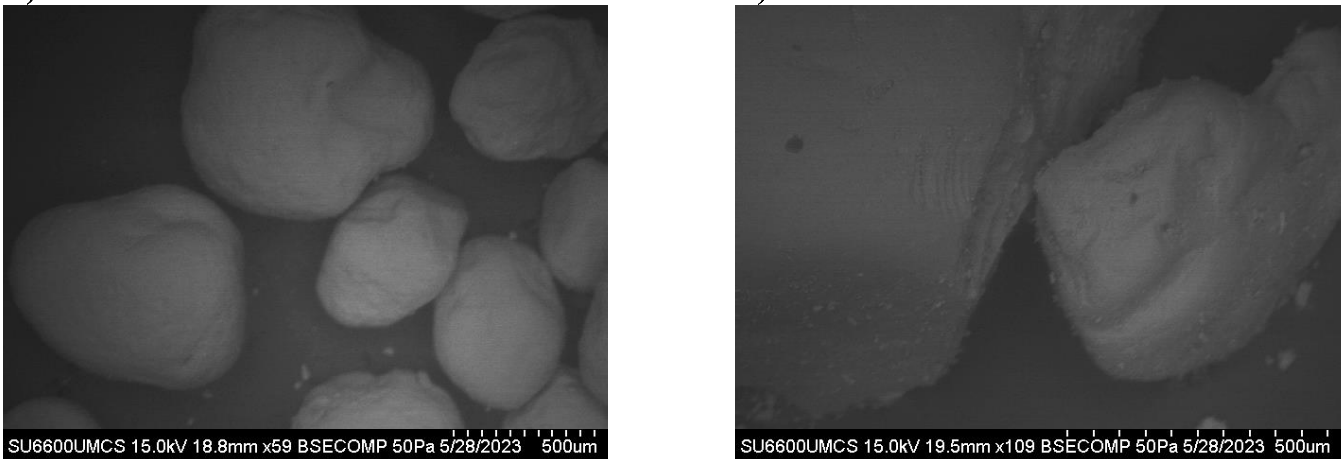

Fig. S4. Image of backscattered electrons (Hitachi SU6600) of sand grains from C horizon of (A) 611 and (B) 805 profiles of Arenosols showing particles adsorbed on the grain surface.

Table S15. Results (%) of micro-area analyzes (obtained using NSS 3.0 and Excel) of sand grains from C horizon of 611 and 805 profiles of Arenosols.

Point	C	O	F	Na	Mg	Al	Si	Cl	K	Ca	Ti	Fe	Ba	Phase on the quartz surface
805(1)_pt1	12.73	43.76			0.55	6.49	28.42		1.54	0.49	2.29	3.72		biotite
805(1)_pt2	24.44	40.39		0.22	0.21	1.89	24.53		0.80			0.90		illite
805(1)_pt3	13.88	43.36			0.42	7.23	32.13		1.93			1.05		illite
805(1)_pt4	10.51	47.20				1.95	39.75		0.60					quartz
805(1)_pt5	8.96	45.11				4.82	34.29		5.86		0.96			illite
805(1)_pt6	10.45	46.72				1.35	40.99		0.49					quartz
805(1)_pt7	30.06	35.74			0.66	1.09	18.31	0.40		3.26				gibbsite
805(1)_pt8	31.71	39.91				3.23	22.19		1.63		1.33			illite
805(1)_pt9	21.96	42.55			0.26	3.09	29.92		1.12			1.11		illite
805(1)_pt10	15.41	40.36			0.23	3.86	37.63		1.50			1.02		illite
805(1)_pt11	13.21	43.56			0.27	4.09	35.23		1.40		1.93	0.30		illite

805(1)_pt12	16.11	43.83				1.25	38.31		0.50					gibbsite
805(2)_pt1	3.67	42.98				9.83	31.41		12.11					orthoclase
805(2)_pt2	2.61	40.03				10.34	32.95		14.07					orthoclase
805(2)_pt3	4.64	38.16		0.30		9.47	32.61		14.82					orthoclase
805(2)_pt4	3.49	41.22				9.70	31.59		14.01					illite
805(2)_pt5	2.67	38.30		0.39		9.29	33.17		16.17					orthoclase
805(2)_pt6	2.20	42.29				4.63	43.90		6.97					quartz
805(2)_pt7	1.91	43.94				3.77	48.00		2.38					quartz
805(2)_pt8	1.87	44.50				2.21	50.40		1.02					quartz
805(2)_pt9	3.63	38.32				2.17	54.94		0.94					quartz
805(2)_pt10	2.43	48.91		1.73		6.94	36.01		1.57		1.00	1.03		illite
805(2)_pt11	3.38	45.10			0.41	5.72	35.96		2.46			6.97		illite + lepidocrocite
805(2)_pt12	3.39	48.43			0.25	2.80	44.13		1.00					quartz
805(2)_pt13	4.21	47.37			0.34	4.60	40.65		1.52			1.31		quartz
805(2)_pt14	2.71	46.38	0.52		0.55	6.75	39.94		3.14					illite
805(2)_pt15	4.24	42.19	0.10			7.46	42.75		3.27					muscovite
805(2)_pt16	7.94	33.86				6.15	47.82		4.23					illite
805(2)_pt17	12.91	40.09					38.04		1.99					quartz
805(2)_pt18	5.28	40.84			0.22	2.80	49.36		1.50					quartz
805(2)_pt19	6.87	38.97				7.79	39.17		7.20					orthoclase
805(2)_pt20	6.98	40.93				3.37	46.98		1.74					quartz
805(2)_pt21	8.38	41.95			0.58	7.50	33.24		3.20		1.24	3.92		illite
805(2)_pt22	7.12	43.03			0.35	4.55	39.39		1.90			1.54	2.13	biotite
805(2)_pt23	10.20	41.89			0.35	4.78	39.13		1.73			1.91		illite
805(2)_pt24	8.22	44.60			0.39	4.88	38.31		1.72			1.88		biotite
805(2)_pt25	9.75	40.97				2.55	45.82		0.90					quartz
805(2)_pt26	12.42	39.13	0.16			4.60	41.96		1.72					illite
805(2)_pt27	5.13	45.97			0.30	4.67	41.86		2.08					illite
805(2)_pt28	12.82	39.78			0.56	10.55	29.27		4.47			2.55		illite
805(2)_pt29	15.01	38.80				8.14	27.44		8.26		2.35			illite
805(2)_pt30	14.03	38.61				9.56	26.66		6.30		1.86	2.98		orthoclase
805(3)_pt1	3.99	44.97				3.37	46.52		1.14			0.01		quartz
805(3)_pt2	3.88	47.04			0.42	4.74	40.23		1.65			2.04		illite
805(3)_pt3	4.01	47.32			0.44	5.73	37.17		1.66			3.66		illite
805(3)_pt4	4.38	44.95			0.64	9.51	31.74		5.00			3.78		illite
805(3)_pt5	4.04	45.42			0.32	3.55	45.32		1.35			0.00		quartz
805(3)_pt6	3.94	47.51			0.39	3.37	43.04		1.08			0.66		quartz
805(3)_pt7	4.30	45.48				3.30	45.81		1.11					quartz
805(3)_pt8	6.19	45.37				3.41	43.96		1.07					quartz
805(3)_pt9	4.70	48.02				1.84	45.44							quartz
805(3)_pt10	4.78	45.92				2.03	46.16		1.10					quartz
805(3)_pt11	20.30	42.30				4.24	33.16							gibbsite
805(3)_pt12	19.26	41.28				4.39	35.07							gibbsite
805(3)_pt13	20.75	39.56				5.06	34.64							gibbsite
805(3)_pt14	18.65	43.11				4.45	33.78							gibbsite
805(3)_pt15	15.79	45.06				4.78	34.37							gibbsite
805(3)_pt16	14.64	42.90				5.14	37.32							gibbsite
805(3)_pt17	17.94	40.78				3.91	31.00							gibbsite
805(3)_pt18	18.50	41.98				3.96	35.55							gibbsite
805(3)_pt19	2.24	45.93	0.30		0.75	9.91	35.47		3.56			1.83		muscovite
805(3)_pt20	2.69	44.67				3.60	47.25		1.79					quartz
805(3)_pt21	3.93	36.25					50.11		1.59			1.58		quartz

805(3)_pt22	4.99	35.58				54.21				quartz
805(3)_pt23	6.01	42.65		0.74	11.33	37.63	1.64			illite
805(3)_pt24	4.78	47.59		0.64	10.89	30.60	2.48		3.03	illite
805(4)_pt1	9.72	42.27			2.11	45.91				quartz
805(4)_pt2	8.13	43.06			1.48	47.32				quartz
805(4)_pt3	7.30	44.20			1.33	47.17				quartz
805(4)_pt4	7.41	47.97			1.34	43.27				quartz
805(4)_pt5	7.24	47.74			0.91	44.12				quartz
805(4)_pt6	12.33	35.68			1.02	50.97				quartz
805(4)_pt7	13.29	38.21			0.87	47.63				quartz
805(4)_pt8	13.31	38.98			1.11	46.59				quartz
805(4)_pt9	13.54	39.52			0.89	46.06				quartz
805(4)_pt10	13.76	37.73			1.46	47.06				quartz
805(4)_pt11	11.89	44.92			2.42	38.92			1.84	gibbsite
805(4)_pt12	15.13	39.79	0.19		1.46	42.52	0.92			quartz
805(4)_pt13	13.67	42.16			1.63	42.53				quartz
805(4)_pt14	11.00	45.45			2.42	41.13				quartz
805(4)_pt15	19.77	39.78			8.04	32.41				gibbsite
611(1)_pt1	12.66	41.89		0.59	4.71	33.48	1.05		5.63	illite
611(1)_pt2	9.95	44.02		0.50	5.81	32.51	1.46	1.35	4.40	illite
611(1)_pt3	10.35	45.85		0.45	4.89	33.88	0.71		3.87	biotite
611(1)_pt4	11.35	45.70			3.23	37.75	0.90	1.07		illite
611(1)_pt5	18.04	43.60			2.45	34.84	1.07			illite
611(1)_pt6	11.43	39.11			2.25	47.20				quartz
611(1)_pt7	10.35	41.51		0.51	5.28	40.67	1.67			illite
611(1)_pt8	12.47	37.84		0.48	5.95	38.53	2.83		1.91	illite
611(1)_pt9	9.56	39.92			5.91	42.47	1.67		0.47	quartz
611(1)_pt10	8.80	41.28		0.30	4.47	39.63	1.70		3.81	illite
611(1)_pt11	7.97	44.18			2.32	45.53				quartz
611(1)_pt12	7.99	43.99	0.42		3.71	43.90				quartz
611(1)_pt13	5.59	43.33				42.54				quartz
611(1)_pt14	5.86	46.61			4.04	43.49				gibbsite
611(1)_pt15	5.31	40.95		1.13	7.13	37.56	3.52		4.40	biotite
611(1)_pt16	6.60	41.58			5.73	40.41	1.45		4.23	quartz
611(1)_pt17	5.87	43.05			4.48	45.36	1.24			illite
611(1)_pt18	10.10	40.15		0.89	8.39	31.71	4.58		4.17	illite
611(1)_pt19	12.00	41.49			2.01	44.51				quartz
611(1)_pt20	10.40	43.24			2.68	43.69			0.00	quartz
611(1)_pt21	8.87	47.74			1.59	41.79				quartz
611(1)_pt22	27.10	41.52			3.05	28.32				gibbsite
611(2)_pt1	23.19	34.06		0.45	3.02	38.30	0.99			illite
611(2)_pt2	23.21	34.07			3.99	35.17			3.57	gibbsite
611(2)_pt3	22.76	33.44			3.41	40.40				quartz
611(2)_pt4	13.15	39.09		0.52	4.64	38.96	1.30		2.34	illite
611(2)_pt5	9.34	42.50		0.25	3.42	41.13	0.64		2.73	quartz
611(2)_pt6	13.00	37.72		0.35	4.88	39.66	1.94		2.45	biotite
611(2)_pt7	7.97	43.68			3.22	42.37	0.81		1.95	quartz
611(2)_pt8	6.72	44.11		0.58	4.54	40.73	1.07		2.25	biotite
611(3)_pt1	20.58	38.29	0.09		3.35	36.53	1.17			illite
611(3)_pt2	20.64	36.60			6.37	27.66	2.87		5.86	illite
611(3)_pt3	36.39	35.30			3.93	22.42	1.97			illite
611(3)_pt4	20.44	39.98			3.57	34.66	1.35			illite

611(3)_pt5	19.56	44.11		0.65		3.56	31.09	1.03			illite
611(3)_pt6	14.00	45.90				1.73	37.79	0.57			illite
611(3)_pt7	13.89	42.20				1.73	40.05	0.59		1.54	quartz
611(3)_pt8	13.09	42.57					36.45	0.93		1.79	quartz
611(3)_pt9	13.27	43.17			0.23	2.45	38.90	0.80		1.18	illite
611(3)_pt10	15.10	42.85			0.38	3.49	34.05	0.87		3.25	illite
611(3)_pt11	18.80	41.93				3.03	33.65	0.96		1.63	illite
611(3)_pt12	17.06	42.33			0.50	5.70	27.79	1.32		5.29	illite
611(3)_pt13	18.67	41.10			0.65	7.27	24.80	2.19	1.57	3.75	illite
611(3)_pt14	21.01	40.56				4.37	30.61	3.45			illite
611(3)_pt15	22.68	39.71	0.15		0.26	3.28	32.86	1.05			illite

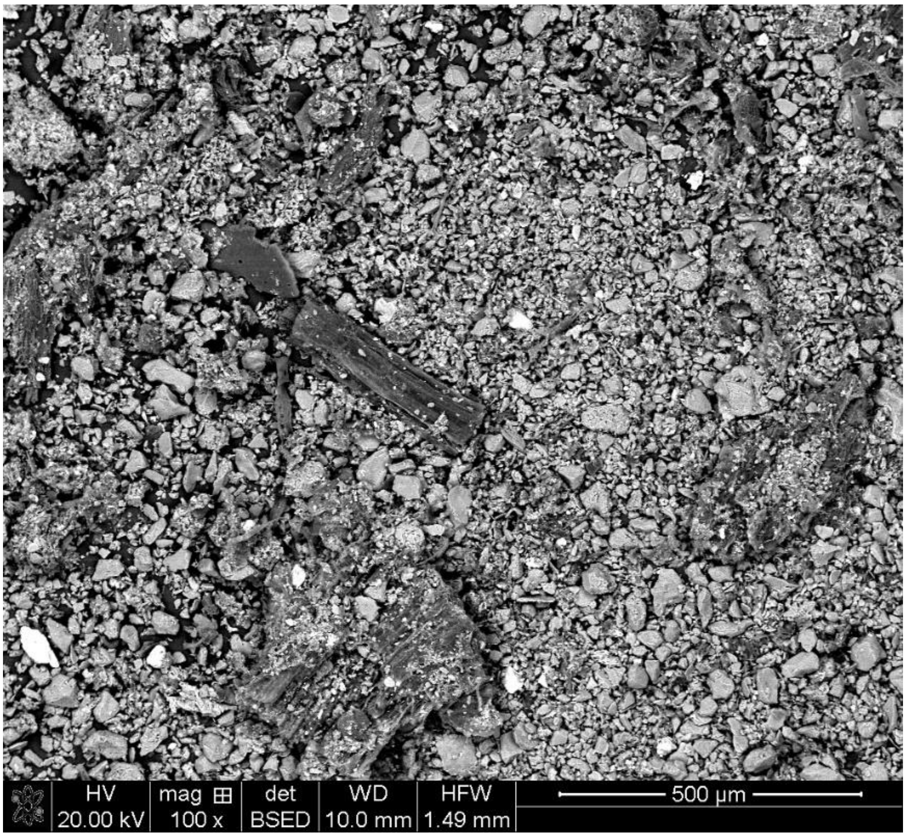

Fig. S5. BSE image of the *HU* 590 Ap sample.

Table S16. Results (%) of micro-area analyzes (obtained using Octane elect Plus and Excel) of *HU* fractions from Ap horizon of 587 and 590 profiles.

Sample	C	N	O	F	Na	Mg	Al	Si	P	S	K	Ca	Ti	Mn	Ni	Cr	Fe	Mineral
587_1-1	55.34	2.55	34.20			0.05	0.78	6.12		0.07	0.24		0.49				0.16	organic
587_1-2	31.34		24.09		0.14	0.38	10.14	18.06			10.28		0.84				4.74	illite
587_1-4	6.32		56.34				0.42	36.92										quartz
587_2-1	9.41		57.08					0.49					32.49				0.53	rutile
587_2-2	8.69		39.40			1.01	10.12	15.06				0.59	0.20	1.23			23.70	goethite
587_2-3	5.44		42.83				0.95	3.33			0.23		46.12			0.17	0.93	rutile
587_2-4	31.01		26.26				0.41	1.96			0.13	0.18	22.92				17.12	ilmenite
587_2-5	14.49		51.37				0.32	33.70					0.13					quartz
587_3-2	70.24	2.34	18.38			0.06	0.78	5.87	0.46	0.64	0.26		0.51				0.48	organic

587_3-4	53.96	3.39	29.74		0.08	0.13	2.00	6.81		0.51	0.95	0.09	1.18		0.74	organic
587_3-5	43.46		33.23			0.25	4.09	12.66	0.63	0.93	1.81	0.41	1.07		1.46	organic
587_4-1	50.48	3.31	26.13			0.19	0.31	19.10		0.12		0.11			0.25	organic
587_5-1	41.78	6.42	29.83		0.24	0.21	4.09	5.75	0.21	0.49	1.26	0.10	8.49		1.14	organic
590_1-1	62.62	3.75	26.20	0.23	0.17	1.38	4.33	0.17	0.50	0.17	0.18	0.31				organic
590_1-2	33.35	2.94	44.25	0.59		0.16	1.92	14.27			0.88	0.23	0.60		0.81	organic
590_1-3	8.71		44.17			1.59	10.30	15.19				3.51		0.76	15.78	chlorite
590_1-4	17.35		47.61					35.04								quartz
590_2-1	61.14	9.23	26.49	0.32	0.08	0.33	0.36	1.39	0.07	0.19	0.11	0.15	0.16			organic
590_2-2	18.85		31.00		0.14		1.13	48.26			0.25		0.38			quartz
590_2-3	16.29	2.58	51.09			1.00	8.91	10.56			1.14	0.25	0.50	0.25	7.39	organic
590_2-4	20.38	8.32	45.32			0.13	2.04	4.21	0.07	0.07	0.47	0.06	12.55	0.36	6.02	organic
590_2-5	25.45	5.03	46.40	0.33		0.28	7.40	9.04	0.07	0.03	2.07	0.00	3.15	0.03	0.71	organic
590_2-6	4.53		7.79				0.78	1.71			0.39		44.77	3.46	36.58	ilmenite
590_3-1	7.13		46.98			0.43	0.18	0.30	0.10				26.40	0.70	17.80	ilmenite
590_3-3	13.28		46.91	2.79			9.28	13.37			0.10	3.82	0.12	1.01	9.41	chlorite
590_3-4	9.37		44.75	1.44				20.69			0.67		20.72		1.87	quartz
590_3-5	4.45		56.30					4.08					35.17			rutile
590_3-6	11.13		17.69				0.59	3.11			0.45	0.55	65.73		0.75	rutile
590_4-1	55.22	6.09	32.43			0.18	1.57	2.89		0.43	0.53		0.35		0.32	organic
590_4-2	40.71		37.24	0.23		0.26	4.68	13.41			2.13	0.06	0.51		0.77	organic
590_5-1	58.02	7.31	30.16			0.09	0.37	3.80		0.14	0.10					organic

S6. Modeling PAAHs adsorption in soils with various pH ranges

The aim of this subsection was to determine which independent variables in Eq. (7) with $FA_{>2.5L}$ and in Eq. (28) (assuming η = 1.0 or 0.1) are significant when the pH of the examined soils is narrower than the examined pH of 4.2−7.7. The modeling results for 12 topsoils and subsoils with pH ≤ 5.6 (n = 36) and 6 topsoils and subsoils with pH ≥ 6.6 (n = 18) were analyzed for this purpose.

In 12 topsoils and subsoils with pH ≤ 5.6, the highest R_a^2 values (0.981−0.993) were obtained with the use of Eq. (7) (denoted as Eq. (S37) in Fig. S6). However, Eq. (S38) produced satisfactory results (R_a^2 of 0.943−0.987). In Eq. (S37) $FA_{>2.5L}$ was regarded as the variable responsible for the adsorption of neutral forms of PAAHs, and only *Al(PA)* and *H(PA)* were regarded as the variables responsible for the adsorption of their anions. A comparison of the results produced by Eqs. (S38) and (S39) revealed that *HA* and *HU* are less important adsorbents of PAAHs anions in acidic soils.

When the *Al(PA)* and *H(PA)* were replaced with $FA_{>2.5L}$, *Al(T)* (f_p was not used – the adsorbents are fully active in acidic soils), and $f_p\ Fe(T)$ (Eq. (S41)), R_a^2 values (0.957−0.988) were comparable to those produced by Eq. (S38). Equation (S42) suggested that the *HU* fraction was also a significant adsorbent of PAAHs anions. The R_a^2 values in Eq. (S42) (0.977−0.993) were comparable to those produced by Eq. (S37). Thus, both the simple Eq. (7), the more complex

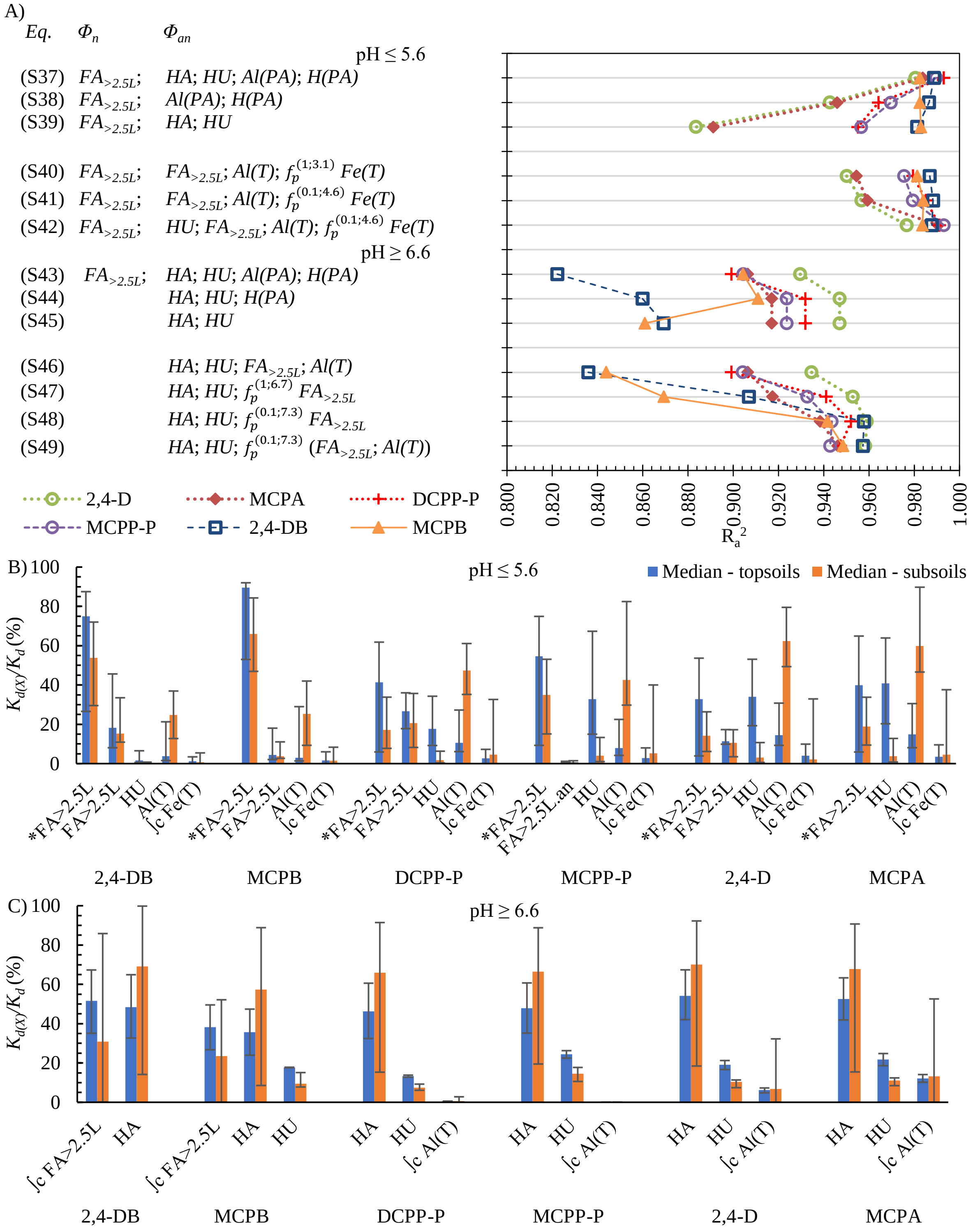

Fig. S6. (A) R_a^2 values for the Lasso regression models on the Y-axis for 12 topsoils and subsoils (n = 36) with pH ≤ 5.6, and 6 topsoils and subsoils (n = 18) with pH ≥ 6.6. (B) Contribution of independent variables from Eq (S42) and (C) from Eq (S49) to the total sorption of PAAHs in the soils with pH ≤ 5.6 and ≥ 6.6, respectively. Error bars – minimum and maximum. Variable with/without * − adsorption of neutral form/anionic form.

Eq. (27), or their simplified forms (Eqs. (S38) or (S41)) can be applied to model of PAAHs adsorption in acidic soils.

In soils with pH ≥ 6.6, only the variables responsible for the adsorption of anions were significant (compare results for Eqs. (S43) and (S44)), including *HA* and *HU*, followed by *H(PA)*. The model with the above variables (Eq. (S44)) produced the best results (R_a^2 values of 0.860-0.947). However, higher R_a^2 values (0.943-0.958) were obtained (Eq.

(S49)) when *H(PA)* was replaced with $f_c\ FA_{>2.5L}$ and $f_c\ Al(T)$, and when η was set to 0.1. The most likely explanation for the above was the low accuracy of the $BaCl_2$-TEA method in determining extractable Al^{3+} and H^+ in neutral or slightly alkaline soils.

The results produced by Eq. (S49) (Fig. S6C) indicate that $f_c\ FA_{>2.5L}$ and *HA* are important adsorbents for 2,4-DB and MCPB anions. The remaining PAAH anions were adsorbed on *HA* and *HU* fractions, and, to a smaller extent, on the sorption sites of Al oxyhydroxides ($f_c\ Al(T)$). Therefore, depending on the examined PAAH, its adsorption at pH > 6.5 can be sufficiently well described using the simplified form of Eq. (S49) with two or three independent variables and with one value of pK_a.

References

Audette Y, Longstaffe JG, Gillespie AW, Smith DS, Voroney RP (2021) Validation and comparisons of NaOH and Na_3PO_4 extraction methods for the characterization of organic amendments. Soil Sci Soc Am J 85:273-285. https://doi.org/10.1002/saj2.20195

Barrett EP, Joyner LG, Halenda PP (1951) The determination of pore volume and area distributions in porous substances. 1. Computations from nitrogen isotherms. J Am Chem Soc 73:373-380. https://doi.org/10.1021/ja01145a126

Bieganowski A, Witkowska-Walczak B, Gliński J, Sokołowska Z, Sławiński C, Brzezińska M, Włodarczyk T (2013) Database of Polish arable mineral soils: a review. Int Agrophys 27:335-350. https://doi.org/10.2478/intag-2013-0003

Cave MR, Harmon K (1997) Determination of trace metal distributions in the iron oxide phases of red bed sandstones by chemometric analysis of whole rock and selective leachate data. Analyst 122:501-512. https://doi.org/10.1039/a607953i

Curtin D, Rostad HPW (1997) Cation exchange and buffer potential of Saskatchewan soils estimated from texture, organic matter and pH. Can J Soil Sci 77:621-626. https://doi.org/10.4141/S97-015

Fox PM, Nico PS, Tfaily MM, Heckman K, Davis J (2017) Characterization of natural organic matter in low-carbon sediments: Extraction and analytical approaches. Org Geochem 114:12-22. https://doi.org/10.1016/j.orggeochem.2017.08.009

Gregor JE, Powell HKJ (1986) Acid pyrophosphate extraction of soil fulvic-acids. J Soil Sci 37:577-585. https://doi.org/10.1111/j.1365-2389.1986.tb00389.x

IHSS (2024) Isolation of IHSS soil fulvic and humic acids. Available online: https://humic-substances.org/isolation-of-ihss-soil-fulvic-and-humic-acids/. Accessed 26 March 2024.

ISO 11260 (2018) Soil quality – Determination of effective cation exchange capacity and base saturation level using barium chloride solution.

ISO 11277 (2020) Soil quality – Determination of particle size distribution in mineral soil material – Method by sieving and sedimentation.

ISO 13536 (1995) Soil quality – Determination of the potential cation exchange capacity and exchangeable cations using barium chloride solution buffered at pH = 8.1.

ISO 14254 (2018) Soil quality – Determination of exchangeable acidity using barium chloride solution as extractant.

Matallo M, Romero E, Sánchez-Rasero F, Peña A, Dios G (1998) Adsorption of mecoprop and dichlorprop on calcareous and organic matter amended soils: Comparative adsorption of racemic and pure enantiomeric forms. J Environ Sci Health B 33:51-66. https://doi.org/10.1080/03601239809373129

OECD (2000) OECD guideline for the testing of chemicals. Method 106. Adsorption-desorption using batch equilibrium method. OECD, Paris

Paszko T (2011) Adsorption and desorption processes of MCPA in Polish mineral soils. J Environ Sci Health B 46:569-580. https://doi.org/10.1080/03601234.2011.586593

Piwowarczyk AA, Holden NM (2013) Phenoxyalkanoic acid herbicide sorption and the effect of co-application in a Haplic Cambisol with contrasting management. Chemosphere 90:535-541. https://doi.org/10.1016/j.chemosphere.2012.08.023

Rumpel C, Rabia N, Derenne S, Quenea K, Eusterhues K, Kögel-Knabner I, Mariotti A (2006) Alteration of soil organic matter following treatment with hydrofluoric acid (HF). Org Geochem 37:1437-1451. https://doi.org/10.1016/j.orggeochem.2006.07.001

Shokrollahi A, Ghaedi M, Niband MS, Rajabi HR (2008) Selective and sensitive spectrophotometric method for determination of sub-micro-molar amounts of aluminium ion. J Hazard Mater 151:642-648. https://doi.org/10.1016/j.jhazmat.2007.06.037

Thorstensen CW, Lode O, Eklo OM, Christiansen A (2001) Sorption of bentazone, dichlorprop, MCPA, and propiconazole in reference soils from Norway. J Environ Qual 30:2046-2052. https://doi.org/10.2134/jeq2001.2046

Wada K, Okamura Y (1977) Measurements of exchange capacities and hydrolysis as means of characterizing cation and anion retentions by soils. Proceedings of the International Seminar on Soil Environment and Fertility

Management in Intensive Agriculture. International Seminar on Soil Environment and Fertility Management in Intensive Agriculture, Soc. Sci. Soil Manure, Japan, pp 811-815

WRB (2015) WRB, World reference base for soil resources 2014, update 2015. International soil classification system for naming soils and creating legends for soil maps. World soil Resources Reports No. 106. FAO, Rome

Xu JS, Zhao BZ, Chu WY, Mao JD, Olk DC, Xin XL, Zhang JB (2017) Altered humin compositions under organic and inorganic fertilization on an intensively cultivated sandy loam soil. Sci Total Environ 601:356-364. https://doi.org/10.1016/j.scitotenv.2017.05.205
